# Does Predation Exacerbate the Risk of Endosymbiont Loss in Heat Stressed Hermatypic Corals? Molecular Cues Provide Insights Into Species-Specific Health Outcomes in a Multi-Stressor Ocean

**DOI:** 10.3389/fphys.2022.801672

**Published:** 2022-03-01

**Authors:** Carolina Madeira, Marta Dias, Ana Ferreira, Raúl Gouveia, Henrique Cabral, Mário S. Diniz, Catarina Vinagre

**Affiliations:** ^1^i4HB – Associate Laboratory Institute for Health and Bioeconomy, NOVA School of Science and Technology, NOVA University Lisbon, Caparica, Portugal; ^2^UCIBIO – Applied Molecular Biosciences Unit, NOVA School of Science and Technology, NOVA University of Lisbon, Caparica, Portugal; ^3^MARE – Marine and Environmental Sciences Centre, Faculty of Sciences, University of Lisbon, Lisbon, Portugal; ^4^Biology Department, Oceanário de Lisboa, Lisbon, Portugal; ^5^INRAE – National Research Institute for Agriculture, Food and Environment, UR EABX, Cestas, France; ^6^CCMAR – Centre of Marine Sciences, University of Algarve, Faro, Portugal

**Keywords:** coral reefs, tissue wounds, ocean warming, predation, molecular responses, biomarkers, health condition, endosymbiont loss

## Abstract

Ocean warming has been a major driver of coral reef bleaching and mass mortality. Coupled to other biotic pressures, corals’ ability for acclimatization and adaptation may become compromised. Here, we tested the combined effects of warming scenarios (26, 30, and 32°C) and predation (wound vs. no wound) in coral health condition (paleness, bleaching, and mortality), cellular stress responses (heat shock protein 70 kDa Hsp70, total ubiquitin Ub, and total antioxidant capacity TAC), and physiological state (integrated biomarker response index, IBR) of seven Scleractinian coral species, after being exposed for 60 days. Results show that although temperature was the main factor driving coral health condition, thermotolerant species (*Galaxea fascicularis*, *Psammocora contigua*, and *Turbinaria reniformis*) displayed increased paleness, bleaching, and mortality in predation treatments at high temperature, whereas thermosensitive species (*Acropora tenuis*, *Echinopora lamellosa*, and *Montipora capricornis* brown and green morphotypes) all died at 32°C, regardless of predation condition. At the molecular level, results show that there were significant main and interactive effects of species, temperature, and predation in the biomarkers assessed. Temperature affected Hsp70, Ub, and TAC, evidencing the role of protein folding and turnover, as well as reactive oxygen species scavenging in heat stress management. Predation increased Hsp70 and Ub, suggesting the activation of the pro-phenoloxidase system and cytokine activity, whereas the combination of both stressors mainly affected TAC during moderate stress and Ub under severe stress, suggesting that redox balance and defense of homeostasis are crucial in tissue repair at high temperature. IBR levels showed an increasing trend at 32°C in predated coral fragments (although non-significant). We conclude that coral responses to the combination of high temperature and predation pressure display high inter-species variability, but these stressors may pose a higher risk of endosymbiont loss, depending on species physiology and stress intensity.

## Introduction

Large-scale bleaching caused by climate change and extreme weather events has become a major theme of coral research in the past years (e.g., Le Nohaïc et al., 2017; Stuart-Smith et al., 2018; Sully et al., 2019; Vargas-Ángel et al., 2019; Hédouin et al., 2020). These bleaching events have caused coral mass mortality in a recent past, with impacts for biogenic reef systems (Hughes et al., 2017, 2018a,b,c), including loss of complex habitat and apex predators, and population explosions of macroalgae that lead to regime shifts (Jackson, 2008; Mumby and Van Woesik, 2014). Based on the current trends of coral reef degradation, the future seems increasingly dire for these critically endangered habitats.

Global climate modeling and satellite observations indicate that the unfavorable thermal conditions that elicit coral bleaching are becoming more prevalent in today’s oceans (Hoegh-Guldberg, 2014; Heron et al., 2016; Liu et al., 2017; IPCC, 2021). Continuous warming of tropical oceans, which are already 1.0 to 1.3°C warmer than in the previous century (Deser et al., 2010), combined with more severe and frequent El Niño events (Power et al., 2013) will continue to aggravate the severity of heat stress suffered by reef corals (Frieler et al., 2013; Ainsworth et al., 2016; Hughes et al., 2018b). Particularly, regions in the Indian and Pacific Oceans where coral reefs occur have shown significant changes in heat content, reflected in the largest increases in surface area with temperatures above 29°C (Lin et al., 2011).

Moreover, warmer seas are likely to drive more intense and frequent tropical storms (Emanuel, 2005; IPCC, 2014, 2021) with an expected increase in the physical damage experienced by coral reefs (Hoegh-Guldberg, 2011). Strong wave action and currents generated during storms, particularly in shallow reefs, are expected to produce an increasing number of coral fragments, through breakage of coral sections from the edges of the colonies (Harmelin-Vivien, 1994). This coral debris can vary in size from small nubbins to fragments as large as 100 cm in length (Highsmith et al., 1980; Forsman et al., 2006), but the increasing magnitude of extreme weather events and consequent mechanical impacts to reefs will likely lead to the generation of increasingly smaller fragments in size. Coupled to evidence from several studies showing that heat stress can negatively affect corals’ sexual reproductive cycles (e.g., Richmond, 1993; Negri et al., 2007; Nozawa and Harrison, 2007; Randall and Szmant, 2009; Levitan et al., 2014), the predicted increases in SSTs may lead to the failure of sexual reproductive processes, which, when coupled to more frequent storms may favor alternative asexual means of propagation, such as coral fragmentation. The detachment and re-distribution of scleractinian corals are important processes in reef systems, as fragmentation leads to colonization of otherwise inaccessible habitats, patch reef development, reef extension, and colony multiplication (Highsmith et al., 1980; Lirman, 2000; Foster et al., 2013; Roth et al., 2013). However, there is a knowledge gap on how and if these small fragments will cope in a multi-stressor environment, and whether they may become key colonizers, allowing for species persistence under climate change.

Evidence from the field and laboratory experiments show that hermatypic coral colonies are overall highly sensitive to heat stress (e.g., see Marshall and Baird, 2000; Hoegh-Guldberg et al., 2007b; Hoegh-Guldberg, 2011; Dias et al., 2018, 2019a), despite species-specific susceptibilities (Dias et al., 2020; McClanahan et al., 2020). Such interspecific variability has been related to corals intrinsic characteristics as well as extrinsic factors. Among intrinsic ones, coral morphology, tissue thickness, metabolic rates, heterotrophic feeding capacity, mucus production rates, and tissue concentration of fluorescent pigments have been shown to play relevant roles in the physiological plasticity of corals and their ability to recover and overcome temperature-induced bleaching (e.g., Salih et al., 1998; Loya et al., 2001; Wooldridge, 2009; Levas et al., 2013; Dias et al., 2018). These intrinsic characteristics can vary with coral size (e.g., full colony vs. large fragment vs. nubbin), affecting coral’s ability to survive under challenging conditions (e.g., see Highsmith et al., 1980; Bowden-Kerby, 2001; Lindahl, 2003). Extrinsic factors include, for instance, the coral location in the reef (i.e., corals in marginal locations are normally exposed to harsher abiotic conditions, see Keshavmurthy et al., 2021).

Eco-physiological studies have provided insights into pathways of coral coping mechanisms through thermal challenges. Authors have highlighted in the literature the importance of Symbiodiniaceae clades to the holobiont thermotolerance (Nesa and Hidaka, 2009; Hillyer et al., 2017; Williams and Patterson, 2020), as well as the relevance of increased abundance of certain proteins (e.g., repertoire of heat shock protein homologs) and antioxidant enzymes that act as intracellular stabilizers (Lesser, 2006). These biomolecules play a role in corals’ cellular stress responses, by controlling macromolecular damage that arises during physiological stress and by scavenging reactive oxygen species (Cziesielski et al., 2019). Nevertheless, the use of these biomolecules as diagnostic biomarkers was only implemented during the early 2000s (Downs et al., 2000; Parkinson et al., 2019).

Since hermatypic corals are the base species of biogenic reef systems, they influence ecosystems’ structure and function (Ellison, 2019), and provide habitats and micro-habitats to a wide range of marine organisms. The importance of predators in maintaining high habitat spatial heterogeneity as well as in structuring benthic communities and has been shown by multiple observational and manipulative studies (Dulvy et al., 2004; Clements and Hay, 2018). Damage by coral predators (i.e., corallivorous organisms), such as butterflyfish, parrotfish, pufferfish, triggerfish, filefish, wrasses, damselfish, *Drupella* snails, and starfish (especially *Acanthaster planci*), range from minor to lethal. The intensity of predation can trigger cascading effects on coral populations (Done et al., 2010; Kayal et al., 2012), as predation causes lacerations to tissues and injuries to the aragonite skeleton that require extensive energetic, molecular, and cellular resources to repair (Rice et al., 2019). Even small wounds on tissue or skeletal structures can cause growth disturbances with fitness consequences (Rotjan and Lewis, 2008), and repeated corallivory can become a source of chronic stress. In fact, outbreaks of both *Drupella* spp. snails and starfish *A. planci*, for instance, have provoked severe biological disturbance of tropical coral reefs throughout the Indo-Pacific oceans (e.g., see Cumming, 2009; Pratchett et al., 2009; Osborne et al., 2011; De’ath et al., 2012; Bruckner et al., 2017). Intense predation by corallivorous organisms has the potential to decimate hermatypic coral communities, thus modifying the physical structure of reefs (Hoeksema et al., 2013; Liu et al., 2017; Moerland et al., 2019). In addition, coral predators also feed preferentially of specific coral genera (e.g., De’ath and Moran, 1998; Pratchett, 2001; Morton et al., 2002), which may affect the composition of reef coral communities.

In this study, we aimed to investigate the combined effects of ocean warming and predation pressure in small coral fragments (mimicking recently re-attached storm-generated fragments) of seven Scleractinian coral species. We assessed corals’ health, bleaching, mortality, and molecular biomarkers, through laboratory experiments. In a recent meta-analysis reviewing the methods of coral heat stress experiments in the past 30 years, although around 47% of the studies included multiple factors in the design, these were essentially abiotic factors (e.g., temperature combined with acidification, light, or nutrients), and only 12% of studies used long-term experiments (≥35 days; McLachlan et al., 2020). Therefore, we will fill the knowledge gap by using a design that combines testing three levels of temperature treatments coupled to two levels of predation pressure for 60 days in small coral fragments. We will identify the most vulnerable species in a post-storm environment under different multi-stressor scenarios, by assessing corals’ physiological condition and ability to survive under the combined stressors.

## Materials and Methods

### Experimental Design and Sampling

This study evaluated seven coral species: *Acropora tenuis* and *Psammocora contigua* with branching morphologies, *Montipora capricornis* brown morphotype (BM), *Turbinaria reniformis*, and *Echinopora lamellosa* with plating morphologies, *M. capricornis* green morphotype (GM) with encrusting morphology, and *Galaxea fascicularis* with massive morphology. Colony morphology designations were based on Marshall and Baird (2000) and Loya et al. (2001). The coral colonies used in the present study were obtained from a stock aquarium at Oceanário de Lisboa (Portugal), with known thermal history for the past 5 years (where they were maintained at a constant temperature of 25°C).

All experiments were also performed at Oceanário de Lisboa, Portugal.^1^ A total of 60 small coral fragments (replicates) for each of the seven species were cut from each mother colony using a pair of pliers or a pincer. Fragments were about 20–40 mm in height for the branching coral colonies, and approximately 30 mm sided squares for the plating, encrusting, and massive corals. Fragment size aimed to mimic small coral fragments potentially generated during tropical storms. Each fragment was placed on the top of a nylon expansion anchor, glued with epoxy putty and properly labeled, to simulate recently re-attached small coral fragments (post-storm). Fragments from branching corals were placed in a vertical position, whereas fragments of corals from the remaining morphologies were placed in a horizontal position. The positioning of the fragments was performed with the aim of minimizing the dead tissue area that resulted from the epoxy putty application. Fragments (mounted in their respective anchors) were allowed to recover from handling for 1 day in the coral stock aquarium over egg crate panels, prior to being transferred to the experiment system.

To determine long-term responses of different coral species to the effects of increased temperatures combined with predation pressure, the coral fragments were exposed to different treatments in a 7 × 3 × 2 factorial design: (a) seven coral species as previously described; (b) three temperatures, including control (26 ± 0.2°C) and elevated temperatures (30 ± 0.5 and 32 ± 0.5°C, respectively); and (c) two predation treatments including control (undamaged coral fragments) and predated corals (single predation wound in coral tissue/polyps).

The experiment design consisted of doing three sequential treatments of 60 days using different coral fragments. The experiment system used in all treatments included one aquarium (400 L), a sump (280 L) with bioballs to improve water quality through biological filtration, a Hailea 500 chiller, and two Fluval M300 heaters to control for temperature. Additionally, a Litpa Jet5 floodlight with an AquaMedic 400 W HQi lamp (13,000 K) ensured appropriate light conditions and was kept on a 12L:12D cycle. To maintain low nutrient concentrations, an AquaMedic Turboflotor 5,000 Shorty protein skimmer was also used. An AquaMedic OceanRunner 3,500 pump was used for water circulation, providing a turnover rate of 12 min. Additionally, an AquaClear 110 powerhead was used to maintain surface water motion. Air-stones were used to maintain high oxygen concentrations.

Each of the sequential treatments consisted of setting the experimental aquarium to 26°C (first treatment), 30°C (second treatment), and 32°C (third treatment) for 60 days. Temperature was increased in each treatment at a 1°C hour^−1^ rate, starting at the temperature of the coral stock aquarium (25°C), allowing for coral fragments to acclimate to the new temperatures for 1, 5, and 7 h, respectively, in each treatment, prior to the experiment start. The selected heating rate mimics temperature changes during spring tides in coral reef-flat communities (Berkelmans and Willis, 1999), where most of the coral species in this study inhabit (Brown and Suharsono, 1990). Within each temperature treatment, *N* = 20 small coral fragments of each species were used. Of these, *N* = 10 fragments species^−1^ were used as predation controls (undamaged), whereas the remaining *N* = 10 fragments species^−1^ were used in the predation groups. In summary, sample sizes included *N* = 10 coral fragments of each species per combination tested (26°C undamaged; 26°C predated; 30°C undamaged; 30°C predated; 32°C undamaged; and 32°C predated); N_species_ = 60 coral fragments; and N_total_ = 420 coral fragments. Corals in the predation treatments were inflicted with circular injuries (3 mm in diameter) to the tissue, designed to simulate damage caused by predators, specifically butterflyfish, which rip off the coral tissue, but do not affect the calcium carbonate skeleton. Injuries were performed with a cutting disk using a Dremel rotatory tool. To avoid edge effects, one injury was inflicted to each replicate fragment, in their middle section. Undamaged and predated coral fragments from the different species were placed over 40 × 40 cm egg crate panels in the same experimental aquarium for each treatment, to better mimic an emerging biodiverse reef and increase ecological realism, as multiple coral species usually co-exist in the wild (Clements and Hay, 2019, 2021). Nevertheless, to avoid unpredictable agonistic inter-species effects, coral fragments of the same species were grouped together, and species were randomly allocated in pairs of parallel rows positioned 2 cm apart from one another (undamaged fragments in one row vs. predated fragments in a parallel row). Egg crates were suspended 15 cm below the water surface of aquaria.

A spheric quantic sensor (LI-193SA) coupled to a data logger (1,400 model) was used to measure Photosynthetically Active Radiation (PAR) levels, which varied between 320 and 345 μE.m^−2^ s^−1^ (400–700 nm waveband). Water quality parameters including temperature, pH (kept at 8.3), and salinity (kept at 33 psu) were measured once a day. Ammonium (kept at 0 mg L^−1^), nitrites (kept between 0.002 and 0.005 mg L^−1^), nitrates (kept between 0 and 2 mg L^−1^), calcium concentration (kept between 389 and 401 mg L^−1^), alkalinity (kept at 100 mg L^−1^), and oxygen concentration (kept between 6.5 and 8.0 mg L^−1^) and oxygen saturation (kept at 104%) were measured once a week. Cleaning routines were performed at least three times a week to avoid algal growth, and these included cleaning the expansion anchors with a toothbrush and replacing egg crates.

### Coral Health Condition Assessments

Health condition of coral fragments was assessed visually according to the following categories: normal, pale, bleached, and dead, as defined by Jokiel and Coles (1974) and Obura (2001). Whereas “normal” corals had healthy tissue and their regular coloration pattern, “pale” corals had visible decreases in their pigmentation, consistent with partial bleaching, but were not white. “Bleached” corals on the other hand had brilliant white or colorless tissue, indicating total loss of endosymbionts, and “dead” corals were devoid of tissue, and only their skeleton remained (i.e., visible corallite structure). Coral health condition was assessed on the last day of the experimental period by the same technician to avoid observer bias. The number of coral fragments fitting into each condition category was counted and transformed into percentage values (%). Afterward, five coral fragments of each species were sampled per treatment combination for biochemical analyses, by removing them from their respective anchor, and placing each fragment inside labeled sterilized tubes on ice-cold conditions. However, there were treatment combinations where no fragments could be sampled at all due to 100% mortality after 60 days of exposure (Table 1) (Overall sample sizes for biomarker assessments were as follows: *N* = 5 fragments species^−1^ per combination tested, when available; N_species_ = 30 coral fragments for each of the three species that survived at 32°C, N_species_ = 20 coral fragments for each of the for four species that died at 32°C; and N_total_ = 170 coral fragments). Samples were then stored at −80°C until further analysis.

**Table 1:** Summary of coral health condition [normal, pale, bleached, and dead (%)] per individual species and grouped species (thermosensitive vs. thermotolerant) and treatment tested at 60 days (N_total_ = 420 coral fragments).

					Condition (%)			
Species	Morphology	Temperature (°C)	Predation	*N*	Normal	Pale	Bleached	Dead
*Acropora tenuis*	Branching	26	no lesion	10	100.0	-	-	-
			with lesion	10	80.0	-	-	20.0
		30	no lesion	10	100.0	-	-	-
			with lesion	10	100.0	-	-	-
		32	no lesion	10	-	-	-	100.0
			with lesion	10	-	-	-	100.0
*Echinopora lamellosa*	Plating	26	no lesion	10	100.0	-	-	-
			with lesion	10	100.0	-	-	-
		30	no lesion	10	100.0	-	-	-
			with lesion	10	100.0	-	-	-
		32	no lesion	10	-	-	-	100.0
			with lesion	10	-	-	-	100.0
*Montipora capricornis* BM	Plating	26	no lesion	10	100.0	-	-	-
			with lesion	10	100.0	-	-	-
		30	no lesion	10	40.0	60.0	-	-
			with lesion	10	10.0	90.0	-	-
		32	no lesion	10	-	-	-	100.0
			with lesion	10	-	-	-	100.0
*M. capricornis* GM	Encrusting	26	no lesion	10	100.0	-	-	-
			with lesion	10	100.0	-	-	-
		30	no lesion	10	100.0	-	-	-
			with lesion	10	100.0	-	-	-
		32	no lesion	10	-	-	-	100.0
			with lesion	10	-	-	-	100.0
Thermosensitive species	Multiple	26	no lesion	40	100.0	-	-	-
			with lesion	40	95.0	-	-	5.0
		30	no lesion	40	85.0	15.0	-	-
			with lesion	40	78.0	23.0	-	-
		32	no lesion	40	-	-	-	100.0
			with lesion	40	-	-	-	100.0
*Galaxea fascicularis*	Massive	26	no lesion	10	100.0	-	-	-
			with lesion	10	100.0	-	-	-
		30	no lesion	10	100.0	-	-	-
			with lesion	10	100.0	-	-	-
		32	no lesion	10	40.0	10.0	50.0	-
			with lesion	10	10.0	20.0	70.0	-
*Psammocora contigua*	Branching	26	no lesion	10	100.0	-	-	-
			with lesion	10	100.0	-	-	-
		30	no lesion	10	37.5	62.5	-	-
			with lesion	10	10.0	90.0	-	-
		32	no lesion	10	-	70.0	-	30.0
			with lesion	10	-	50.0	-	50.0
*Turbinaria reniformis*	Plating	26	no lesion	10	100.0	-	-	-
			with lesion	10	100.0	-	-	-
		30	no lesion	10	9.1	90.9	-	-
			with lesion	10	-	100.0	-	-
		32	no lesion	10	-	100.0	-	-
			with lesion	10	-	100.0	-	-
Thermotolerant species	Multiple	26	no lesion	40	100.0	-	-	
			with lesion	40	100.0	-	-	
		30	no lesion	40	49.0	51.0	-	
			with lesion	40	37.0	63.0	-	
		32	no lesion	40	13.0	60.0	17.0	10.0
			with lesion	40	3.0	57.0	23.0	17.0

Thermosensitive species include *A. tenuis*, *E. lamellosa*, and *M. capricornis* BM and GM; thermotolerant species include *G. fascicularis*, *P. contigua*, and *T. reniformis*.

### Biomarkers

Scleractinian coral samples were briefly washed with ultrapure water to clean debris. Samples were then crushed using a mortar and pestle and the mixture of crushed tissue and aragonite were transferred to microtubes and further homogenized in 1 ml of phosphate buffer saline solution (PBS, pH 7.4) on ice. Homogenized samples were centrifuged for 15 min at 10,000 × *g* at 4°C. Supernatants were stored ultra-frozen at −60°C.

Total proteins were quantified using the Bradford assay (Bradford, 1976). Briefly, sample duplicates were added to microplate wells (96-well polystyrene microplates from Greiner, Austria) to which Bradford reagent (from Bio-Rad, United States) was added and absorbance was read at 595 nm in a microplate reader (Synergy HTX, BioTek, Winooski, VT, United States). Protein concentration was calculated from a calibration curve of 0.0 to 2.0 mg ml^−1^ constructed from bovine serum albumin (BSA) standards.

Stress biomarkers heat shock protein 70 kDa (Hsp70), total ubiquitin (Ub), and total antioxidant capacity (TAC) were then quantified. These biomarkers were selected due to their important roles in cellular stress responses of organisms to environmental challenges and thermal adaptation: (i) Hsp70 is a chaperone that stabilizes degrading proteins (Jolly et al., 2018); (ii) Ub signals irreversibly damaged proteins for degradation in the proteasome (Madeira et al., 2014) and TAC quantifies both enzymatic and non-enzymatic antioxidants, which indicates the ability to counteract oxidative stress-induced damage in cells (Padmini and Geetha, 2009).

Hsp70 and Ub were both quantified through indirect enzyme linked immunosorbent assays (ELISA; see Madeira et al., 2017). Briefly, for each of these biomarkers, duplicates of each sample were pipetted into microplates wells (96-well high-binding microplates from Greiner, Austria) and incubated overnight at 4°C. On the following day, microplates were washed three times with PBS 0.05% Tween-20, blocked with 1% BSA in PBS, and incubated at 37°C for 90 min. After microplate washing (as described previously), primary monoclonal antibodies anti-Hsp70/Hsc70 (OriGene, Germany) and anti-Ub (Abcam, United Kingdom) were diluted to 1.0 μg ml^−1^ in 1% BSA in PBS and were added to microplates wells, respectively. Microplates were then incubated at 37°C for 90 min, followed by another washing step. The secondary antibody (anti-mouse IgG, fab specific, and alkaline phosphatase conjugate, Sigma-Aldrich, United States) was used in both Hsp70 and Ub protocols—it was diluted to 1.0 μg ml^−1^ in 1% BSA in PBS and added to each microplate well, followed by another incubation step at 37°C for 90 min. Microplates were then washed again and substrate was added (SIGMA FAST p-Nitrophenyl Phosphate Tablets, Sigma-Aldrich, United States), and incubated at room temperature for 30 min. Finally, a stop solution (3 M NaOH) was added to each well to stop the reaction and absorbance was read at 405 nm in a microplate reader (Synergy HTX, BioTek, Winooski, VT, United States). Hsp70 and Ub concentrations were then calculated using calibration curves constructed from serial dilutions of purified Hsp70 active protein (OriGene, Germany) and of purified Ub (UbpBIO, United States) standards, respectively, to give a range of 0.0 to 2.0 mg ml^−1^.

TAC assay followed procedures developed by Miller et al. (1993) and Kambayashi et al. (2009). Duplicates of each sample were added to microplates wells (96-well polystyrene microplates from Greiner, Austria). Then, 90 μM myoglobin in phosphate buffer 50 mM pH 8.0 was added to each well, followed by 600 μM ABTS [2,2′-Azino-bis (3-ethylbenzthiazoline-6-sulfonic acid)] and 500 μM hydrogen peroxide. Microplates were incubated at room temperature for 5 min and absorbance was read at 410 nm. Trolox (6-hydroxy-2,5,7,8-tetramethylchroman-2-carboxylic acid) standards were used to produce a calibration curve from 0.0 to 0.330 mM, then used for TAC concentration calculations.

Biomarker values were normalized per total proteins in each sample (μg mg^−1^ for Hsp70 and Ub, and mM mg^−1^ for TAC).

### Data Analyses

Data matrices were first tested for assumptions of normality and homoscedasticity with Shapiro’s Wilk and Levene’s test, respectively. When assumptions were rejected, data were log-transformed and auto-scaled (i.e., mean centered and divided by the standard deviation, SD).

Coral health condition datasets for individual species were analyzed as percentages, and a MANOVA was then employed to test for effects of (i) temperature and predation, as well as their interactive effects in overall health condition of all coral species combined; whereas Student’s t-tests were used to (ii) test the effects of species thermal sensitivity (thermotolerant vs. thermosensitive) in paleness, bleaching, and mortality, using Statistica v8 (Statsoft, United States).

To explore biomarker data structure, multivariate analyses were employed. Principal component analysis (PCA) was performed based on group separation within each independent categorical variable (species, temperature, and predation), followed by heatmaps constructed based on hierarchical cluster analysis of biomarkers, using Euclidean distance and complete linkage algorithm as metrics. Metaboanalyst v5.0 online software was used to run these analyses (https://www.metaboanalyst.ca/, Chong et al., 2019).

To identify the main effects and interactions of variables (i) species, (ii) temperature, and (iii) predation in biomarkers, multi-factorial MANOVAs and ANOVAs were employed. Given that our design was incomplete due to 100% mortality of four species at 32°C, for which biomarker measurements are missing in that treatment, we performed two separate sets of tests: one for species that survived moderate stress (30°C, all seven species) and another one for species that also survived severe stress (32°C, three species).

Integrated biomarker indices were then calculated for each species and stress conditions, following the methodology described in our previous papers (Madeira et al., 2018; Dias et al., 2020). First, the overall mean (m) and the standard deviation (s) for each biomarker were calculated and standardized (Y) as Y = (X − m)/s, where X is the mean value for the biomarker at a given experimental condition. Afterward, Z was calculated using Z = −Y or Z = Y, in the case of biomarker inhibitions or stimulations, respectively. The score (S) was calculated by S = Z + |Min|, where S ≥ 0 and |Min| is the absolute value for the minimum of all calculated Y for a given biomarker. Star plots were then used to display score results (S) and to calculate the IBR as:


$$IBR=\overset{n}{\underset{i=1}{\sum }}Ai$$

$$Ai=\frac{Si}{2}\sin\beta \left(Si\;\cos\beta +S_{i+1}+\sin\beta \right)$$

$$\beta =\tan^{-1}\frac{S_{i+1}\sin\alpha \;}{Si-S_{i+1}\cos\alpha }$$

where Si and Si + 1 are two consecutive scores (radius coordinates and clockwise direction) of a given star plot; Ai is the area connecting two scores; n is the number of biomarkers used for calculations; and *α* = 2п/n. To identify temperature and predation main and interactive effects in Scleractinian corals’ IBRs (joining all species), a factorial ANOVA was employed.

## Results

**Figure 1:**
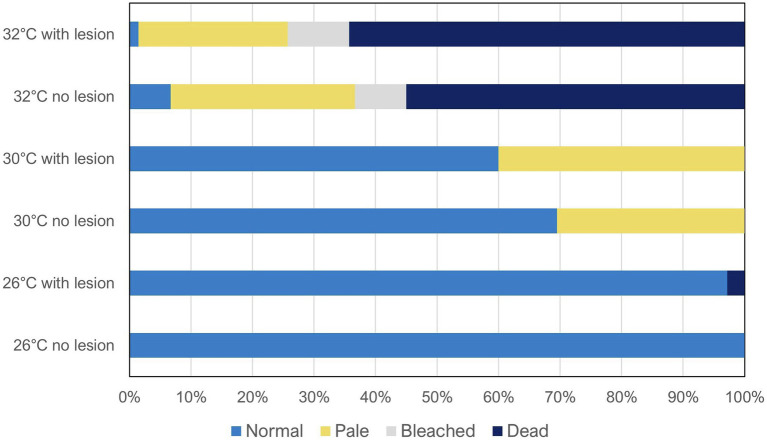
Coral reef health condition (normal, pale, bleached, and dead) after 60 days of exposure to the different treatments (temperatures: 26 vs. 30 vs. 32°C; predation: no lesion vs. with wound lesion). The graph shows average percentage values for all species combined (*N* = 60 fragments per species, N_total_ = 420 coral fragments).

Coral health condition results are presented in Table 1 and Figure 1. The most temperature-sensitive species were *A. tenuis*, *E. lamellosa*, and both morphotypes of *M. capricornis* (hereinafter defined as thermosensitive), which displayed 100% mortality after 60 days at 32°C, independently of predation conditions (Table 1). The effects of temperature alone were also seen in the remaining species *G. fascicularis*, *P. contigua*, and *T. reniformis*, which were able to withstand the 32°C treatment (hereinafter defined as thermotolerant); however, *P. contigua* displayed 30% mortality and *G. fascicularis* showed 50% bleaching (in undamaged, non-wounded corals), besides increased paleness. Even though all species tolerated 60 days at 30°C, *M. capricornis* BM, *T. reniformis*, and *P. contigua* also displayed increased paleness at this temperature (Table 1). Changes in coral condition upon predation at control temperature occurred only in *A. tenuis* (20% mortality). Additionally, decreases in coral health under the combination of elevated temperature and predation only occurred in four species: (i) *M. capricornis* BM at 30°C—paleness increased from 60% in undamaged fragments to 90% in wounded fragments, (ii) *G. fascicularis* at 32°C—paleness and bleaching increased from 10 to 20% and from 50 to 70%, respectively, in undamaged and wounded fragments, (iii) *P. contigua* at 30 and 32°C—paleness increased from 62.5 to 90% (30°C) and mortality increased from 30 to 50% (32°C) in undamaged vs. wounded fragments, and finally (iv) *T. reniformis* at 30°C—paleness increased from 90.9 to 100% in undamaged vs. wounded fragments (Table 1). Moreover, species thermal sensitivity (thermosensitive vs. thermotolerant) was found to have an effect in coral paleness and mortality under the tested multi-stressors (Student’s *t*-tests, paleness *t* = −3.18, value of *p* = 0.002, mortality *t* = 2.56, value of *p* = 0.014).

Overall, when analyzing all species combined (Figure 1), temperature was found to be the main factor significantly affecting coral health condition (Wilks’ test, *F* = 18.99, value of *p* < 0.0001), whereas predation alone was not significant (Wilks’ test, *F* = 0.23, value of *p* = 0.873). Despite the also non-significant interaction between temperature and predation in health condition of all species combined (Wilks’ test, *F* = 0.055, value of *p* = 0.999), as high inter-species variability was observed, there still seems to be a trend of decreased condition with predation in some individual species, specifically at 30 and 32°C, as previously described (Table 1).

**Figure 2:**
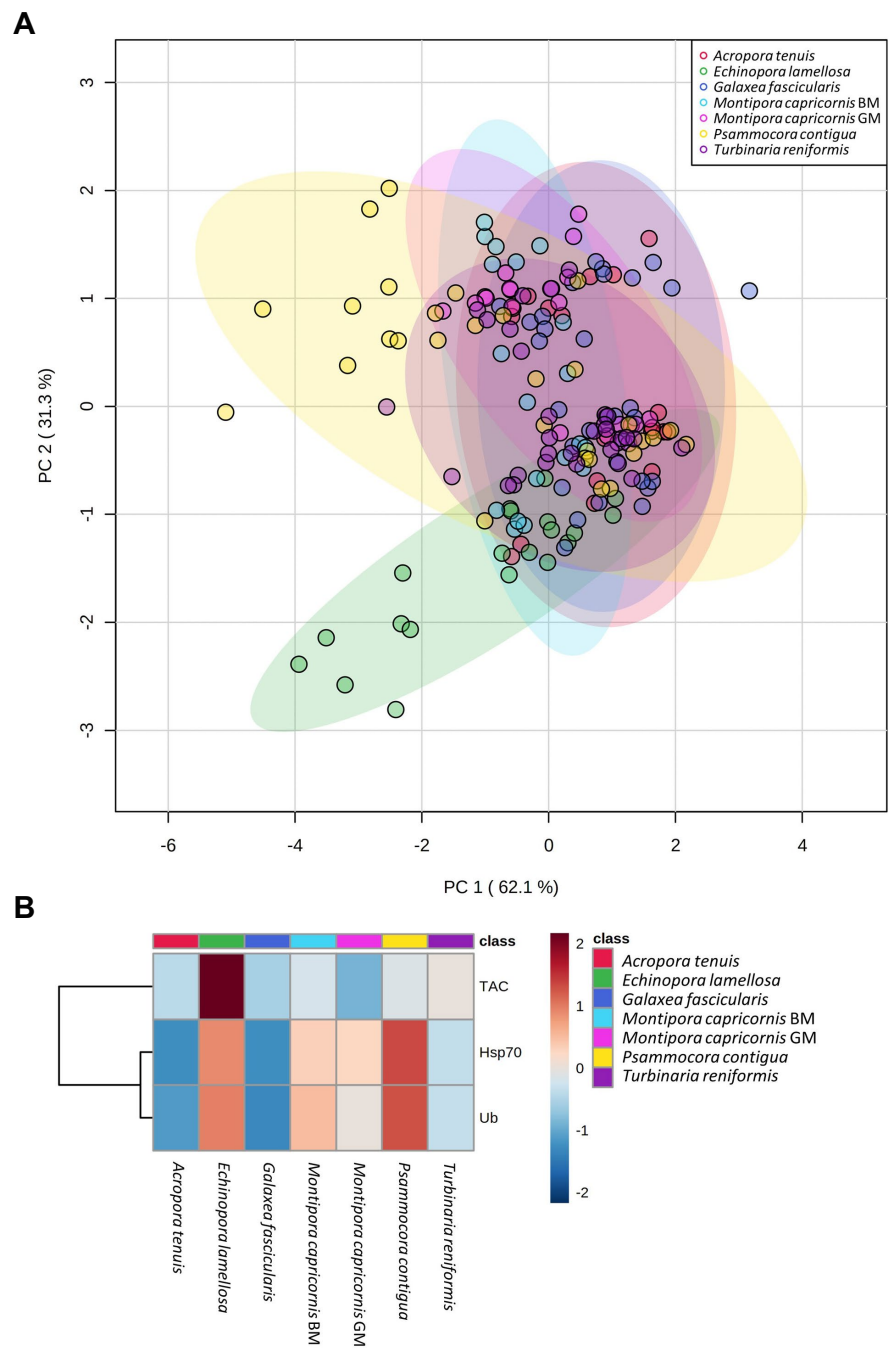
Differences in biomarker profiles of seven Scleractinian coral species tested in this study. **(A)** PCA score plot of biomarker profiles; **(B)** heatmap of clustered biomarkers; in the color scale, red indicates higher than mean concentration, and blue indicates lower than mean concentration. Rows are biomarker concentrations and columns are group averages. Significant differences were found for biomarker profiles between species (Table 2).

**Figure 3:**
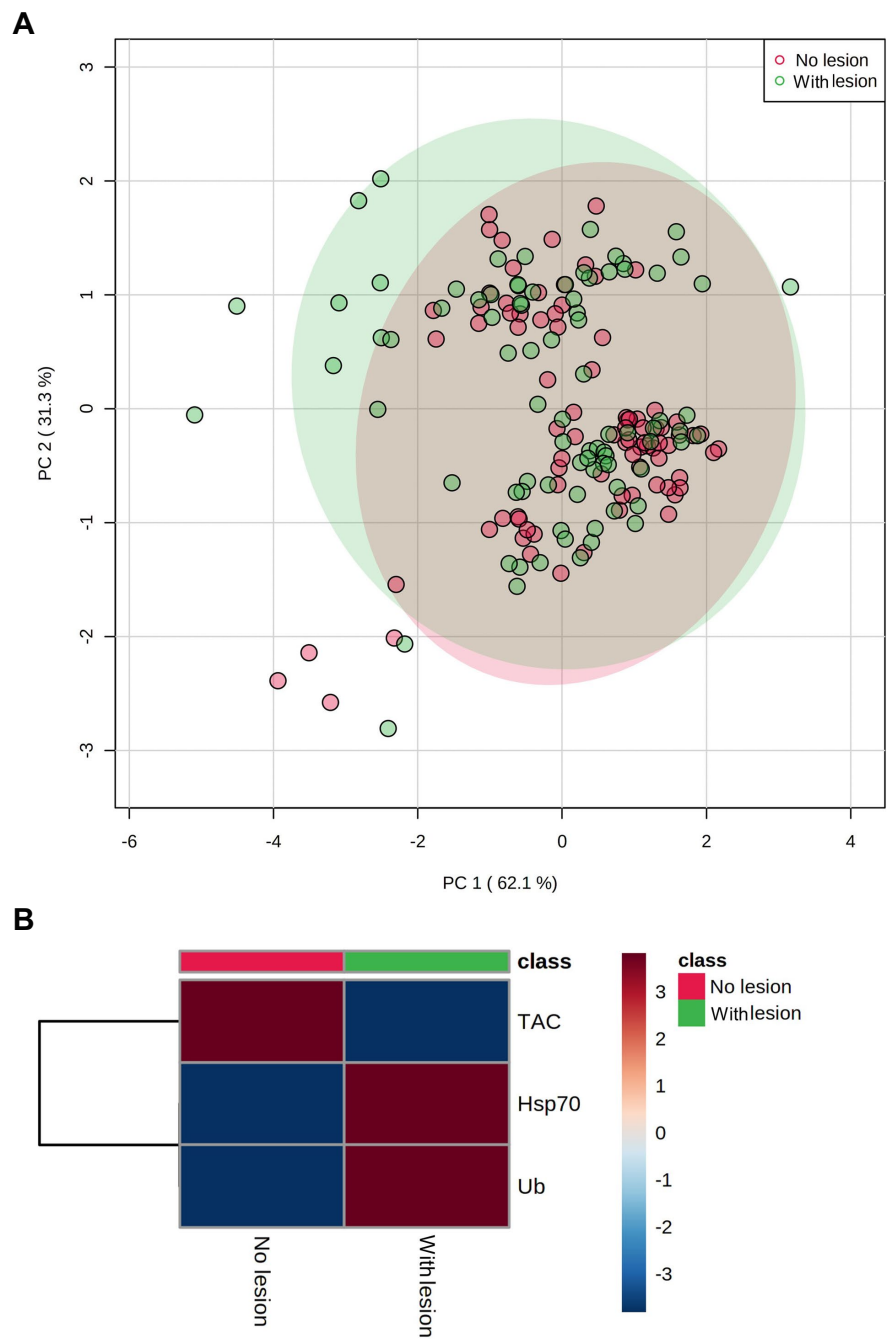
Differences in biomarker profiles of corals with tissue wound lesions from predation vs. no tissue lesions. **(A)** PCA score plot of biomarker profiles; **(B)** heatmap of clustered biomarkers; in the color scale, red indicates higher than mean concentration, and blue indicates lower than mean concentration. Rows are biomarker concentrations and columns are group averages. Significant differences were found for biomarker profiles between predation conditions (Table 2).

**Figure 4:**
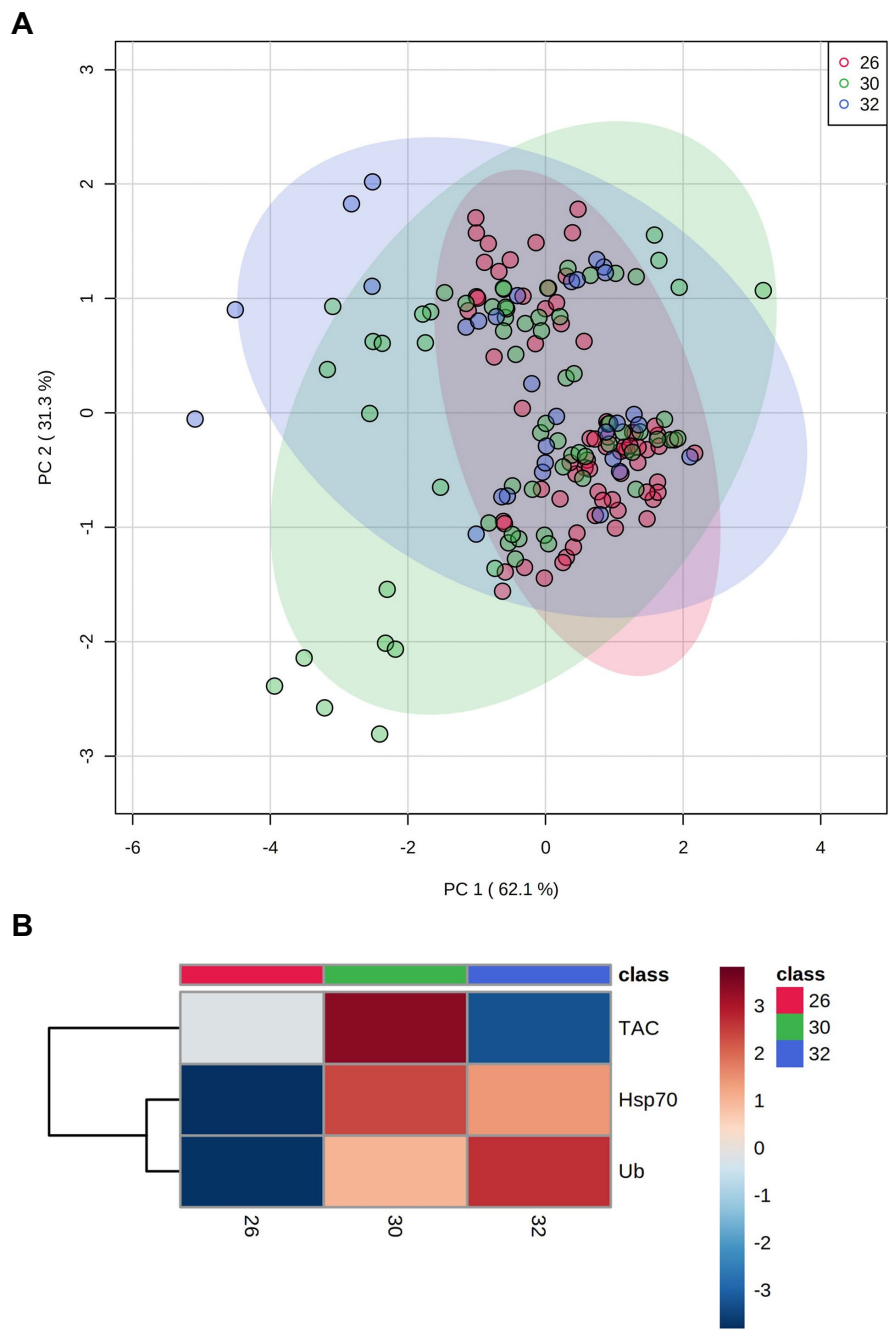
Differences in biomarker profiles of corals exposed to three temperature conditions: 26 vs. 30 vs. 32°C. **(A)** PCA score plot of biomarker profiles; **(B)** heatmap of clustered biomarkers; in the color scale, red indicates higher than mean concentration, and blue indicates lower than mean concentration. Rows are biomarker concentrations and columns are group averages. Significant differences were found for biomarker profiles between temperature conditions (Table 2).

PCA results plotting biomarkers by independent factors showed an explained variance of 62.1% for PC1 and 31.3% for PC2, yielding a cumulative value of 93.4% for the first two components. PCA 2D scores plot for different species (Figure 2) showed a certain degree of overlap between them; however, there was a distinction between *E. lamellosa* and *P. contigua* clusters from the remaining species. Heatmap results further evidenced these differences, showing that *E. lamellosa* displays higher overall concentrations of TAC, whereas *P. contigua* displays higher overall concentrations of Hsp70 and Ub, when compared to the remaining species. PCA 2D scores plot for predation conditions (Figure 3), in turn, showed overlap between specimens with a wound lesion vs. no lesion; however, specimens with wound lesions displayed higher variance in stress responses. The heatmap showed that specimens with no lesion had higher TAC, which decreased under predation. In contrast, Hsp70 and Ub concentrations generally increased in specimens with predation wounds. Finally, PCA 2D scores plot for temperature differences (Figure 4) showed that specimens exposed to 26°C were grouped in a small cluster, whereas specimens exposed to both 30 and 32°C were grouped in larger clusters with higher variability in responses. Heatmap results showed that all biomarker concentrations were relatively low at 26°C, but all increased at 30°C (especially TAC and Hsp70), and then at 32°C, two different trends were observed: TAC and Hsp70 concentrations decreased, but Ub concentrations increased further, in comparison with the previous temperature conditions.

Results from multivariate factorial MANOVAs (Table 2) showed that all stressors (species, predation, and temperature) and their interactions had a significant effect in the tested biomarkers, both in species that survived moderate thermal stress (30°C, all species), as well as severe thermal stress (32°C, *G. fascicularis*, *P. contigua*, and *T. reniformis*). Univariate factorial ANOVAs (Table 2) showed significant differences for Hsp70 for factor “species” and “temperature” as well as interactions “species × predation” “species × temperature,” and “species × predation × temperature” in corals that survived moderate stress (Table 2A). Moreover, corals that survived severe stress showed significant differences in Hsp70 concentration for all factors and their combinations tested, except for “predation × temperature” (Table 2B). Ub showed identical results to Hsp70, with the exception for “predation × temperature” for species that survived severe stress, which was also significant (Table 2B). Results for TAC concentrations in species that survived moderate stress showed that both “predation” and “temperature” alone did not significantly affect this biomarker; however, all factors’ interactions showed significant effects (Table 2A). For species that survived severe stress, factors “species” and “temperature,” as well as interactions “species × predation” and “species × temperature” showed significant effects in TAC concentrations (Table 2B). Overall, the results of this table show that all biomarkers were different between species, that predation only affected Hsp70 and Ub and that temperature affected all biomarkers. All factor interactions also affected the tested biomarkers, and the most relevant interaction in the context of this study—“predation × temperature,” differentially affected species that survived moderate stress (TAC levels) and species that survived severe stress (Ub levels), respectively (Table 2). See also supplemental material for post-hoc Tukey HSD tests for ANOVAs (Supplementary Tables SM1–SM9; Supplementary Figures SM1–SM7).

**Table 2:** Factorial MANOVAs and ANOVAs testing the main and interactive effects of (i) species, (ii) predation, and (iii) temperature in biomarkers Hsp70, Ub, and TAC concentrations: (A) for coral species that survived up to 30°C (all seven species); (B) for coral species that also survived at 32°C (thermotolerant *Galaxea fascicularis*, *Psammocora contigua,* and *Turbinaria reniformis*).

			Species	Predation	Temperature	Species × Predation	Species × Temperature	Predation × Temperature	Species × Predation × Temperature
A	Multivariate	λ	0.217	0.874	0.710	0.615	0.315	0.903	0.543
		F	12.356	5.272	14.957	3.240	8.729	3.926	4.162
		*p*-value	<0.001^*^	0.001^*^	<0.001^*^	<0.001^*^	<0.001^*^	0.010^*^	<0.001^*^
	Hsp70	MS	2.702	0.027	6.681	2.593	3.355	0.214	3.408
		F	5.986	0.060	14.801	5.745	7.431	0.473	7.550
		*p*-value	<0.001^*^	0.806	<0.001^*^	<0.001^*^	<0.001^*^	0.493	<0.001^*^
	Ub	MS	2.405	2.816	14.535	1.776	3.763	0.112	1.163
		F	7.093	8.303	42.861	5.238	11.096	0.331	3.430
		*p*-value	<0.001^*^	0.005^*^	<0.001^*^	<0.001^*^	<0.001^*^	0.566	0.004^*^
	TAC	MS	12.113	0.228	1.040	0.785	5.030	3.190	0.788
		F	41.801	0.787	3.590	2.710	17.360	11.009	2.721
		*p*-value	<0.001^*^	0.377	0.061	0.017^*^	<0.001^*^	0.001^*^	0.017^*^
B	Multivariate	λ	0.473	0.628	0.365	0.640	0.395	0.790	0.630
		F	10.568	13.815	15.280	5.832	6.491	2.907	2.948
		*p*-value	<0.001^*^	<0.001^*^	<0.001^*^	<0.001^*^	<0.001^*^	0.010^*^	<0.001^*^
	Hsp70	MS	9.740	4.967	5.197	4.681	2.872	1.272	3.923
		F	19.109	9.746	10.197	9.185	5.634	2.495	7.696
		*p*-value	<0.001^*^	0.003^*^	<0.001^*^	<0.001^*^	0.001^*^	0.090	<0.001^*^
	Ub	MS	8.626	12.638	15.074	3.170	5.658	1.924	0.922
		F	28.884	42.315	50.472	10.615	18.946	6.441	3.086
		*p*-value	<0.001^*^	<0.001^*^	<0.001^*^	<0.001^*^	<0.001^*^	0.003^*^	0.021^*^
	TAC	MS	1.512	0.000	1.919	1.717	1.121	0.874	0.639
		F	4.667	0.001	5.922	5.300	3.459	2.699	1.973
		*p*-value	0.012^*^	0.974	0.004^*^	0.007^*^	0.012^*^	0.074	0.108

Significant results (value of *p* < 0.05) are presented with an asterisk (^*^).

**Figure 5:**
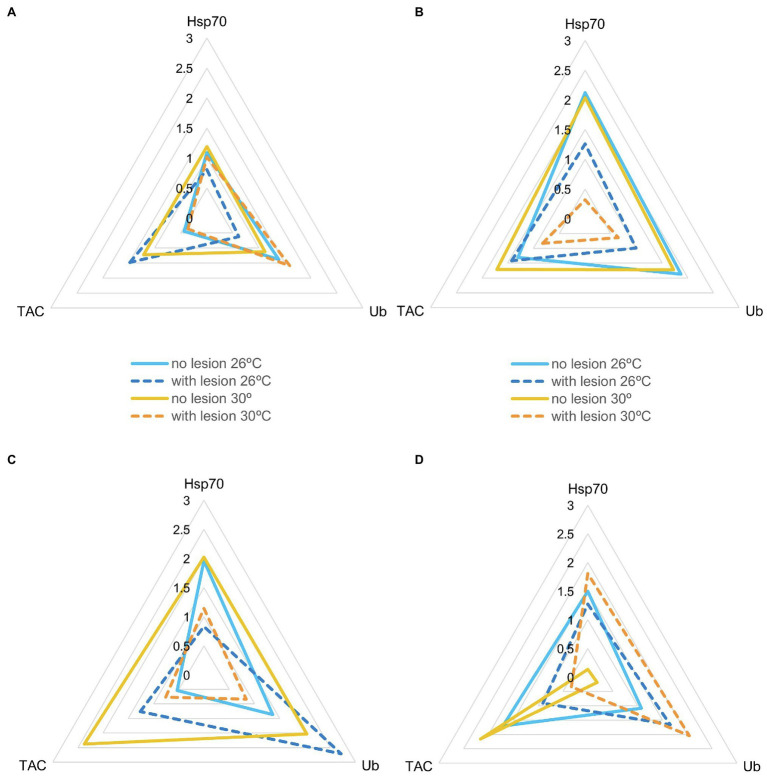
Star plots with mean scores obtained in each experimental condition for thermosensitive coral species that only survived up to 30°C (N_total_ = 20 fragments per species, *N* = 5 specimens of each species per experimental condition). **(A)** *Acropora tenuis* (IBR_26 + no lesion_ = 1.29; IBR_26 + lesion_ = 1.30; IBR_30 + no lesion_ = 2.07; IBR_30 + lesion_ = 1.28); **(B)** *Echinopora lamellosa* (IBR_26 + no lesion_ = 4.58; IBR_26 + lesion_ = 2.22; IBR_30 + no lesion_ = 4.97; IBR_30 + lesion_ = 0.49); **(C)** *Montipora capricornis* BM (IBR_26 + no lesion_ = 2.21; IBR_26 + lesion_ = 3.37; IBR_30 + no lesion_ = 6.86; IBR_30 + lesion_ = 1.22); and **(D)** *M. capricornis* GM (IBR_26 + no lesion_ = 3.00; IBR_26 + lesion_ = 2.38; IBR_30 + no lesion_ = 0.35; IBR_30 + lesion_ = 2.48).

**Figure 6:**
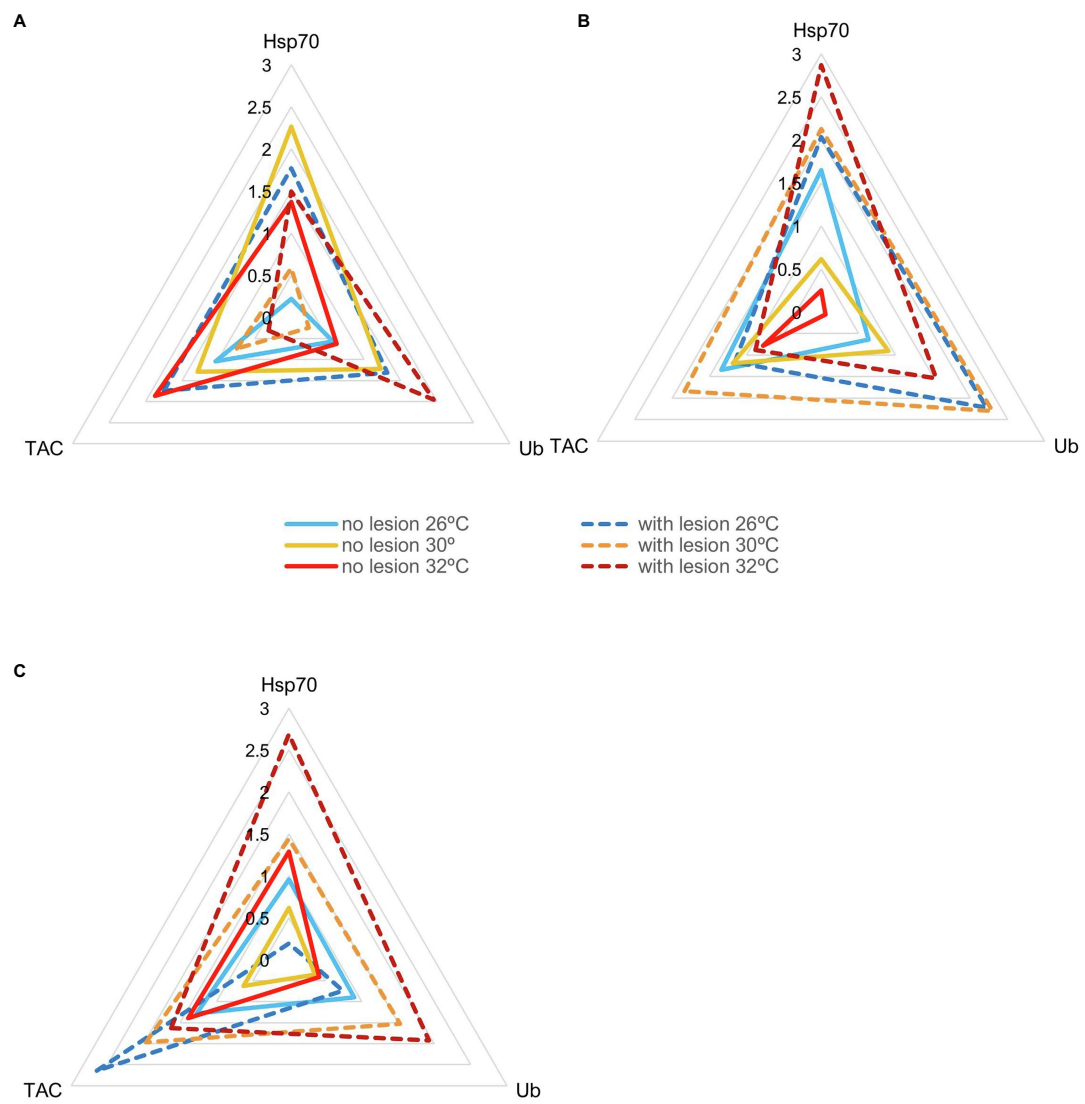
Star plots with mean scores obtained in each experimental condition for thermotolerant coral species that survived up to 32°C (N_total_ = 30 fragments per species, *N* = 5 specimens of each species per experimental condition). **(A)** *Galaxea fascicularis* (IBR_26 + no lesion_ = 0.47; IBR_26 + lesion_ = 3.85; IBR_30 + no lesion_ = 3.63; IBR_30 + lesion_ = 0.37, IBR_32 + no lesion_ = 2.28; IBR_32 + lesion_ = 1.99); **(B)** *Psammocora contigua* (IBR_26 + no lesion_ = 2.05; IBR_26 + lesion_ = 4.74; IBR_30 + no lesion_ = 1.17; IBR_30 + lesion_ = 6.50, IBR_32 + no lesion_ = 0.13; IBR_32 + lesion_ = 4.16); and **(C)** *Turbinaria reniformis* (IBR_26 + no lesion_ = 1.60; IBR_26 + lesion_ = 1.30; IBR_30 + no lesion_ = 0.41; IBR_30 + lesion_ = 4.03, IBR_32 + no lesion_ = 1.44; IBR_32 + lesion_ = 6.34).

**Figure 7:**
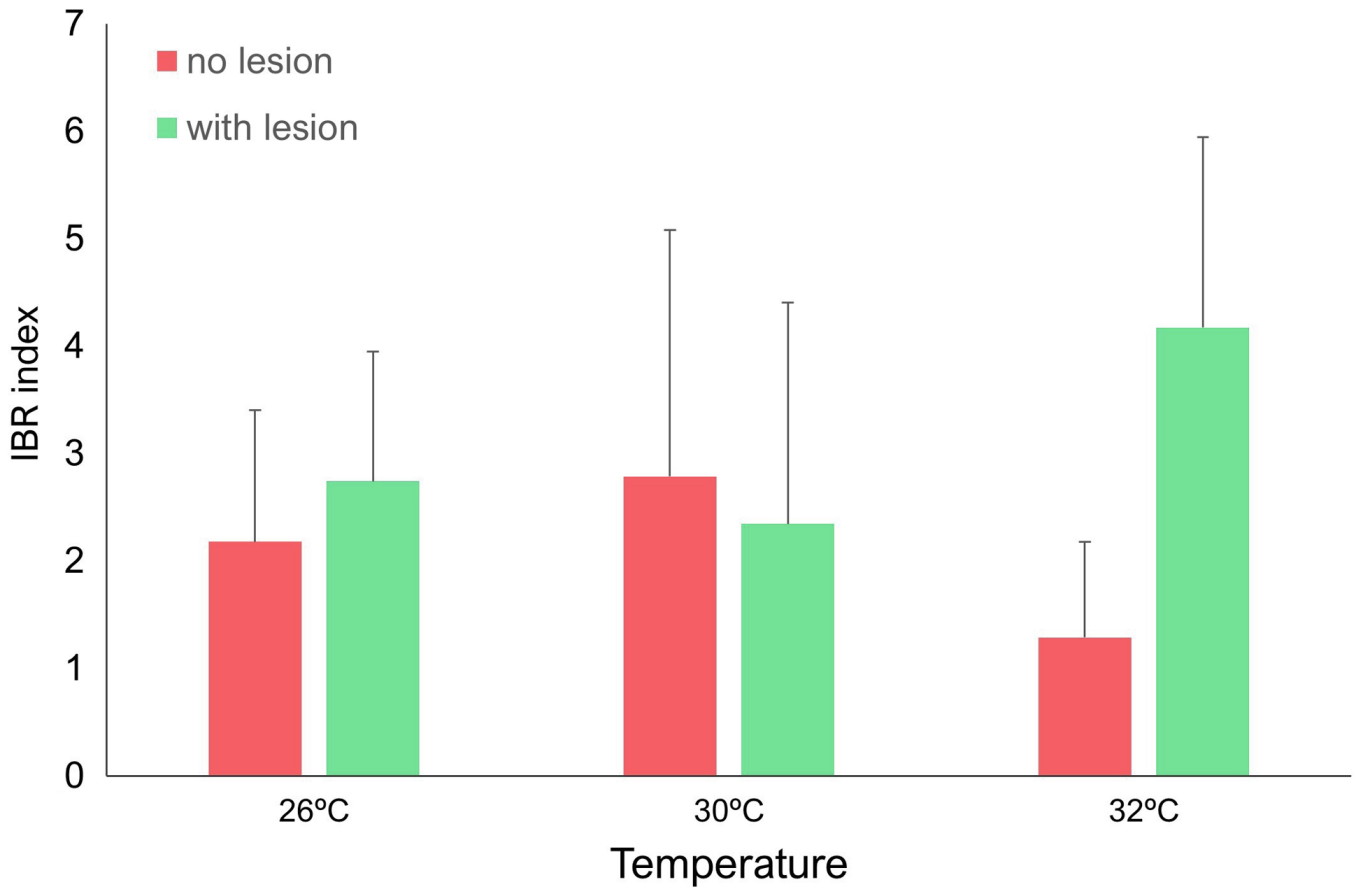
IBR (Integrated Biomarker Response) index (mean + sd of the seven coral species) for all temperatures tested (26, 30, and 32°C) under different predation treatments (no lesion vs. with wound lesion in coral fragments).

IBR results are shown in Figures 5–7 (and Supplemental Material, Supplementary Figures SM8–SM12). Overall, changes in IBRs with temperature and predation were highly variable between species. For coral fragments without predation lesions, increases in IBRs with temperature were observed for *A. tenuis*, *M. capricornis* BM, and *G. fascicularis* (from 26 to 30°C, Figures 5A,C, 6A) and for *T. reniformis* (from 30 to 32°C, Figure 6C), whereas decreases were observed for *M. capricornis* GM from 26 to 30°C (Figure 5D), *T. reniformis* from 26 to 30°C (Figure 6C), and *G. fascicularis* as well as *P. contigua* from 30 to 32°C (Figures 6A,B). For coral fragments with predation lesions, an increase in IBR with temperature was mostly evident for *T. reniformis* (Figure 6C, both at 30 and 32°C) and *P. contigua* (at 30°C, Figure 6B), whereas decreases were observed for *E. lamellosa* and *M. capricornis* BM (Figures 5B,C). Results from the factorial ANOVA testing the effects of both temperature and predation in IBR values for all coral species combined showed, however, no significant differences for any of the factors or interactions assessed (temperature, *F* = 0.04, value of *p* = 0.957; predation, *F* = 2.06, value of *p* = 0.161; and temperature × predation, *F* = 1.641, value of *p* = 0.212). Nevertheless, Figure 7 shows that for corals exposed to 32°C, there was an increasing trend in IBR when comparing undamaged fragments to fragments with predation lesions. Moreover, for predated coral fragments, mean IBR at 32°C was generally trending higher than at 30 or 26°C.

## Discussion

In this study, we investigated how small fragments of hermatypic coral species were affected by ocean warming combined with predation wounds. Specifically, we investigated changes in overall corals’ health condition, namely, paleness, bleaching, and mortality, as well as in molecular responses of Hsp70, Ub, and TAC, and assessed whether the combined stressors had additive or synergistic effects in the tested species.

First, our results showed that under favorable temperature conditions (i.e., control treatment), almost 100% of small coral fragments from the different species not only survived but maintained a healthy pigmentation throughout the experiment, even under predation conditions. This finding suggests that storm-generated fragments can contribute to species persistence in the wild, despite their reduced size, if the post-storm abiotic environment is appropriate. Additionally, low intensity predation alone also does not seem to be a determining factor in small coral fragments’ health outcomes (except for *A. tenuis* that displayed 20% mortality in predated fragments at control temperature), suggesting that they can resist the pressure of agonistic biotic interactions early after re-attachment. These results contradict earlier hypothesis of coral fragment size-dependent survivorship, which proposes that the percentage of fragments surviving is a power function of fragment size (in cm; see Highsmith et al., 1980). Research from the last decades has also found opposing trends to this hypothesis. For instance, Lewis (1991) did not find size-dependent survival of *Millepora complanata* fragments along fringing reefs in Barbados, West Indies. Forsman et al. (2006) on the other hand found that the occurrence of size-specific mortality in coral nubbins of *Porites lobata* and *P. compressa* from Hawaii depends on the *ex-situ* nursery conditions.

Second, we found that small coral fragments potential contribution to species persistence may be compromised under future ocean warming conditions. Our results overall show that high temperatures decreased small coral fragments’ health condition after 60 days of exposure, as corroborated by other studies with coral colonies (e.g., Weis, 2010; Fujise et al., 2014; Zinke et al., 2015). Increased coral paleness was already visible at 30°C for three species in our experiment, suggesting some degree of zooxanthellae loss and/or photosynthetic pigments degradation (Dias et al., 2018), whereas at 32°C, not only paleness increased further, but bleaching reached values as high as 50% in one species and mortality ranged from 30 to 100%, in the remaining species. The previous result shows that the magnitude of temperature effects is species-specific, but impacts can be consistently expected across the Scleractinia order under ocean warming. Previous studies have already shown that coral species (including colonies and fragments) have different susceptibilities to heat stress and tolerance to bleaching (Marshall and Baird, 2000; Weis, 2010; Pandolfi et al., 2011; Dias et al., 2018; Hughes et al., 2018c). For instance, Gates and Edmunds (1999) suggested that massive corals with high metabolic rates that constrain the quick conversion of energy into body mass and thus have low growth rates acclimatize more efficiently. Conversely, branching species that have a low maintenance metabolism and therefore high growth rates as they can allocate more energy for production are more thermally sensitive (see also Steyermark, 2002 on the relationship between basal metabolism and growth in animals). Hongo and Yamano (2013) also showed that some branching coral colonies, such as *Acropora* spp. from clear-water reefs, cannot tolerate severe thermal stress, whereas massive coral colonies are generally more tolerant. Morikawa and Palumbi (2019) on their turn found that the massive species *Porites cylindrica* had the greatest heat tolerance when compared to acroporid corals *Acropora hyacinthus* (tabular morphology) and *Acropora gemmifera* and the branching *Pocillopora damicornis*. On the other hand, we found that coral fragments of *A. tenuis*, together with *E. lamellosa* and *M. capricornis* brown and green morphotypes (the former with branching and the two latter with plating and encrusting morphologies), were intolerant to 32°C, displaying 100% mortality after 60 days of exposure. In contrast, the massive species in our study *G. fascicularis* and one other plating species *T. reniformis* were the only ones that did not display any mortality at the highest temperature. This last observation suggests a high degree of thermotolerance and ability to sustain prolonged stress, as Marshall and Baird (2000) had also observed for these two genera (*Galaxea* and *Turbinaria*) in the Great Barrier Reef during field surveys in March 1998, after a mass bleaching event. Although coral condition was only assessed at the end of the experiment in the present study, our observations throughout the experiment suggest that small coral fragments that were dead at 60 days had suffered previous bleaching. As Matsuda et al. (2020) proposed, coral bleaching susceptibility can therefore be a good predictor of subsequent mortality within species but not between coral species. Ecologically, this likely means that future community composition of novel coral reefs emerging from storm-induced asexual fragmentation may become dominated by thermotolerant species in ocean areas with faster warming rates (Smith et al., 2013).

Third, our results failed to identify a common and/or generalizable effect of combined temperature and predation in overall Scleractinian species’ health condition. This was mainly due to high interspecific variability. However, it is also possible that the use of an experimental design that mixed the seven coral species in the experiment tanks may have helped corals to perform better under the multi-stressors, especially under moderate stress (e.g., 30°C combined with predation). Indeed, multiple coral species co-exist in a biodiverse reef and mixed species assemblages have been shown to enhance growth and tissue survivorship, as stated by Clements and Hay (2019, 2021). Nevertheless, our condition assessments of individual species still suggest that some corals, when experiencing heat stress, may be disproportionately negatively affected by the additional pressure caused by fish predators, such as browsers that feed on coral mucus and tissue, as simulated in our experiment. This assertion seems to hold true for at least four species tested, namely, *M. capricornis* BM and *T. reniformis* at 30°C, *P. contigua* at 30 and 32°C, and *G. fascicularis* at 32°C. Changes in health condition in these cases were mainly reflected by an increase in the percentage of pale fragments (all four species), and to a smaller extent on the percentage of bleached and dead fragments (only observed in *G. fascicularis* and *P. contigua*, respectively). Therefore, we suggest that in these cases, an agonistic interaction with predators in a temperature-challenging environment may pose a higher risk of endosymbiont loss. Moreover, these effects seem to occur at different onset temperatures for each species, which suggests a correlation with species heat susceptibility. However, observations of decreases in coral health under elevated temperature and predation are lost once the temperature is too high and induces mass mortality, at least in thermosensitive species. Previous studies have shown relatable findings; for instance, Clements and Hay (2018) showed that coral predators may suppress the resilience of certain coral foundation species especially in stressed or degraded reefs and that selective coral mortality may occur upon high predator densities (Pratchett et al., 2009). Although we did not assess coral growth or fitness in this study, previous authors have also reported decreases in both parameters upon corallivory (Rotjan and Lewis, 2008; Rice et al., 2019), as well as negative population growth if the prey population size is small, even in healthy corals (Dulvy et al., 2004). Nevertheless, more experiments are needed to quantify the effects of different types of corallivore animals on reef corals, given that we did not test deeper lesions, such as the ones caused by scrapers (e.g., parrotfishes) or by excavators (e.g., pufferfishes)—in which skeleton layers/pieces are also removed, together with tissue and mucus (Rice et al., 2019). This would likely have caused higher coral mortality levels in the predation treatment groups after 60 days. Also, testing different predator abundances (e.g., testing one wound lesion vs. multiple wound lesions) would also help us understand and predict coral species trajectories upon predator outbreaks.

Fourth, even though molecular processes involved in heat stress and bleaching are similar among hermatypic corals, there are variations in mechanisms that may lead to different outcomes (Carballo-Bolaños et al., 2020). Results from molecular biomarker analyses in this study showed that temperature significantly affected all biomarkers tested, *via* changes in heat shock proteins, protein ubiquitination, and reactive oxygen species scavengers (the latter only in species that survived the 32°C treatment). These results suggest two things: (i) protein folding, metabolism, and turnover are essential acclimatization mechanisms to elevated temperatures in biogenic corals, as also stated by Gates and Edmunds (1999) and (ii) the inability of thermosensitive species to induce antioxidant responses even during moderate stress (30°C) may explain their mass mortality at 32°C, likely due to potential macromolecular damage and accumulation of cytotoxic by-products derived from oxidative stress (e.g., lipid peroxides, see studies by Lesser, 2006; Ayala et al., 2014 and Dias et al., 2019a). Moreover, Hsp70 and Ub were also key biomarkers in responses to tissue damage caused by predation. The involvement of heat shock-related proteins in the immune system of corals has been previously reported (e.g., for *Acropora millepora*, Brown et al., 2013) and is thought to be related to the activation of the pro-phenoloxidase (ProPO) system in invertebrates (Baruah et al., 2010). This system is linked to invertebrate cytokine activity (Mak and Saunders, 2006), and cytokines are known to regulate the wound-healing cascade (Ottaviani et al., 2004). Moreover, the ProPO system is responsible for the production of melanin and several intermediates which are toxic to pathogens and enhance phagocytosis, avoiding infection of wounded tissues (Brown et al., 2013).

Fifth, the combination of temperature and predation differentially affected molecular responses of corals, which may explain why at a higher biological organization level (whole fragment health condition), changes were observed in some species but not in others, and why we got an overall insignificant result for adverse health outcomes. In species that survived moderate thermal stress, the interaction of both factors resulted in a reduction in TAC levels, vs. in species that survived severe thermal stress, in which an increase in Ub levels was observed when temperature and predation were combined. This suggests that different cellular stress mechanisms are involved in responses to multi-stressors depending on species inherent physiologies and stress intensity. A reduction in TAC levels during exposure to the combined stressors could indicate, in thermosensitive species, the exhaustion of molecular defensive systems and could explain an increase in percentage of paleness in predated fragments at 30°C (e.g., as observed in *M. capricornis* BM). This would occur due to an increase in oxidative stress caused by less effective ROS scavenging, which leads to photosynthetic dysfunction of endosymbionts and damage to their cell compartments (Curran and Barnard, 2021). In consequence, coral hosts probably digested and/or expelled damaged *Symbiodinium* (see Fujise et al., 2014) at higher rates, leading to changes in corals’ pigmentation levels. Similarly, an increase in total ubiquitin levels in thermotolerant corals during exposure to severe thermal stress and predation combined would be expected given that apoptosis regulatory components (including ubiquitin) function primarily as coordinators of tissue repair and remodeling, to regulate the defense of homeostasis, and only secondarily function as cell death inducers, when everything else fails (see Meier et al., 2015). Nevertheless, tissue regeneration is an energetically expensive process (Henry and Hart, 2005), as multiple wound-healing mechanisms are activated, such as TOLL-like pathways to fight pathogens, melanin synthesis for tissue regeneration, apoptosis of damaged cells, and re-distribution of amoebocytes to the wound site (Meszaros and Bigger, 1999; Kramarsky-Winter and Loya, 2000; DuBuc et al., 2014; Rice et al., 2019). This usually requires the transfer of resources between coral polyps, from healthy tissues to wound sites for healing (Rice et al., 2019). Given that skeleton porosity and polyp connection may vary between species, and available resources are limited in small coral fragments, this may explain why thermotolerant species like *G. fascicularis*, *P. contigua*, and *T. reniformis* still displayed variable increases in percentage of paleness, bleaching, and mortality after 60 days of exposure to temperature and predation. Alternatively, Palmer et al. (2011) also found that *Symbiodinium* density was significantly lower in freshly injured tissues when compared to coral healthy tissue, which could trigger molecular and physiological cascades in corals that are already loosing symbionts and becoming pale or bleached due to thermal stress, such as in our experiment. As Meesters and Rolf (1993) pointed out, bleached corals indeed present reduced tissue regeneration potential, increasing colony vulnerability. This may be a determinant factor in coral survival, as healing rates will differ between species, according to their physiology, thermotolerance, and phenotypes (Traylor-Knowles, 2016).

Sixth, previous studies from our team had also found that the combination of elevated temperature (30 and 32°C) with other stressors (e.g., low salinity) induced molecular changes in Scleractinian corals, indirectly revealing oxidative stress, measured by an increase in antioxidant enzymes’ activities, and macromolecular damage, namely, lipid peroxidation (see Dias et al., 2019a,b). Cellular stress responses are fairly conserved among organisms, and similar changes have also been reported for corals exposed to multiple global change stressors in the Mediterranean Sea (Franzellitti et al., 2018), the Red Sea (Seveso et al., 2020), the Maldives (Seveso et al., 2018), the Great Barrier Reef (Hoegh-Guldberg et al., 2007a), the Caribbean (Robbart et al., 2004; Desalvo et al., 2008; Dimos et al., 2019), and altogether, from all tropical latitudes (e.g., see Downs et al., 2000; Edge et al., 2013; Olsen et al., 2013; Seveso et al., 2014; Tisthammer et al., 2021).

Seventh, we found that different coral species employ specific physiological strategies to deal with environmental stress. *E. lamellosa* and *P. contigua* employed a similar molecular strategy: both displayed highly inducible cellular stress responses (CSR) upon stress exposure. However, *P. contigua* had higher overall concentrations of Hsp70 and Ub, when compared to *E. lamellosa*, which had, in contrast, higher overall TAC concentrations. Given that *E. lamellosa* did not tolerate temperatures above 30°C, whereas *P. contigua* did (despite decreased condition in some coral fragments, especially when combined with predation), this suggests that (i) antioxidants *per se* were not enough to prevent mortality induced by extreme oxidative stress and that (ii) molecular chaperones, ubiquitin, and protein regulation may be key to increase survival under unfavorable conditions. These latter biomarkers are, for instance, crucial to prevent the occurrence of cytotoxic protein aggregations in cells (Iwama et al., 1999; Tomanek and Somero, 1999; Sørensen et al., 2003). Notwithstanding, this cannot be raised as a general mechanism, given that it was only observed in these two species. On the other hand, other species, namely, *M. capricornis* (both morphotypes) employed a different molecular strategy, by having high basal/constitutive biomarker expression levels, even in control conditions, which facilitate a faster reaction to mild and moderate stress (e.g., 30°C and/or occasional predation) but were not enough to cope with extreme stress (e.g., 32°C), because their responses were overall less inducible, which is probably explained by energetic trade-offs that exhaust the available defense mechanisms or that prevent an increase in synthesis of protective enzymes and proteins to save energy and molecular resources, as metabolic costs accelerate with high temperatures in ectotherms (Angilletta et al., 2003; Van Der Meer, 2006; Huey and Kingsolver, 2019). This strategy of constitutively expressing a set of protective proteins and enzymes has also been proposed for other corals (*A. hyacinthus*, Barshis et al., 2013) and has been observed in other marine sessile cnidarians, such as sea anemones (Madeira et al., 2019), and invertebrates from environments with low thermal inertia, such as tidal pools (e.g., limpet *Cellana toreuma* Dong and Williams, 2011; *Palaemon longirostris*, *P. elegans*, and *Carcinus maenas*, Madeira et al., 2012). The most stress-tolerant species like *G. fascicularis* and *T. reniformis*, however, showed more oscillation in molecular responses, including bi-phasical responses (i.e., several increases followed by decreases in biomarker levels or *vice-versa*). This suggests that these organisms display considerable molecular flexibility and can quickly acclimatize to changing conditions, which explains their overall tolerance to stress. In future studies, histological analysis of coral samples would be useful to anchor molecular results at higher biological complexity levels and identify tissue changes and pathological processes contributing to thermosensitivity (for a detailed review of histological methods in aquatic organisms, see Costa, 2017; for coral sampling techniques for histological analysis, see Greene et al., 2020).

Eighth, we highlight here that interspecific differences in physiological strategies and molecular responses found in this study are not a product of different recent thermal histories, as this potential source of variability was minimized by using mother coral colonies raised under controlled aquarium temperature in the previous 5 years of the experiment (25°C). This ensured that coral fragments used in this study were not pre-conditioned to neither short- nor long-term thermal stress, which could have caused corals to shift their thermal thresholds and increase tolerance (Brown et al., 2002; Heron et al., 2016; Keshavmurthy et al., 2021).

Lastly, albeit overall IBRs showed non-significant differences under the tested stressors, both separately as well as their interactions, there was a noteworthy trend evidencing that IBR still displayed increasing values for combined effects of high temperature with predation wounds, suggesting higher stress levels. In fact, our previous studies on reef corals indicate that IBRs are generally responsive to increasing temperatures and depend on the coral species (Dias et al., 2020). This is not exclusive of corals, as similar results have been found for other tropical organisms, from fish to crustaceans and gastropods (Madeira et al., 2018). Therefore, the trend found for IBR values under combined stressors is unsurprising. If we transpose these results into a real-word, multi-stressor scenario, in which there are repeated events of thermal stress as well as corallivory, recurrent bleaching, variable wound sizes (in-depth, perimeter, and location), and multiple wounds occurring simultaneously which need longer healing times (Cróquer et al., 2002); then, coral condition and organism ability to respond to multiple interacting stressors may be significantly affected, to the point of becoming compromised (Henry and Hart, 2005). Moreover, coral wounds can be the starting point for disease, especially in thermally stressful environments, during which disease outbreaks increase (Tracy et al., 2019), such as black-band disease or white-band disease (Mayer and Donnelly, 2013; Contardi et al., 2020). Therefore, the combination of these stressors could facilitate the spread of disease vectors (Porter and Tougas, 2001). Altogether, the combination of temperature and predation showed that stressor interaction effects in corals are highly variable among the Scleractinia order, and impacts may be detected only at the molecular level in some species, whereas in a few others, stressors will synergistically interact to decrease coral heath condition. Whether this will ultimately affect reef structure, composition and dynamics remains to be tested.

## Conclusion

Altogether, we consider the main take-home messages from this study to be: (i) the majority of the tested coral species was already suffering from heat stress at 30°C; (ii) thermosensitive species (*A. tenuis*, *E. lamellosa*, and *M. capricornis* BM and GM) displayed mass mortality at 32°C; (iii) under control temperature conditions, most species tolerated predation quite well, maintaining healthy coloration; (iv) predation, when combined with high temperature, increased the risk of endosymbiont loss and/or pigment degradation, mainly in thermotolerant species (*G. fascicularis*, *P. contigua*, and *T. reniformis*); (v) thermotolerant corals presented different morphologies but similar molecular flexibilities which allowed them to acclimate and increase survival odds under stress; and (vi) despite physiological plasticity of thermotolerant corals during stress exposure, this may not always mean that coral resilience will be re-defined by acclimatization, especially if multi-stressors are at play. We can therefore conclude that delving into the inherent variability underlying organisms’ physiology and survival, and “exploring life in context,” as pertinently posed by Heard (2022) seems crucial to understand the dimensions of climate change impacts on biodiversity.

## Data Availability Statement

The datasets presented in this study can be found in online repositories. The names of the repository/repositories and accession number(s) can be found at: doi: 10.5281/zenodo.5831999.

## Author Contributions

CV, HC, and MSD designed the study and supervised the work. AF and RG supervised the experiments. MSD supervised the lab work and MD did the experiments. CM did the lab work and the statistical analyses. CM and MD drafted the paper. All authors critically revised the paper, agreed to the author order, approved the final manuscript version, and agreed to be accountable for the work and take responsibility for accuracy and integrity of the research conducted.

## Funding

Fundação para a Ciência e Tecnologia (FCT, I.P.) funded this study through the project WarmingWebs PTDC/MAR-EST/2141/2012 and strategic projects UIDB/04292/2020 awarded to MARE, UIDB/04326/2020 awarded to CCMAR, and UIDB/04378/2020 and UIDP/04378/2020 awarded to UCIBIO, as well as LA/P/0140/2020 awarded to i4HB. CM was supported by a researcher grant CEECIND/01526/2018, and MD was supported by a PhD grant SFRH/BD/103047/2014, both awarded by FCT. This publication was also funded by the European Union’s Horizon 2020 Research and Innovation Programme under grant agreement N810139: Project Portugal Twinning for Innovation and Excellence in Marine Science and Earth Observation—PORTWIMS.

## Conflict of Interest

The authors declare that the research was conducted in the absence of any commercial or financial relationships that could be construed as a potential conflict of interest.

## Publisher’s Note

All claims expressed in this article are solely those of the authors and do not necessarily represent those of their affiliated organizations, or those of the publisher, the editors and the reviewers. Any product that may be evaluated in this article, or claim that may be made by its manufacturer, is not guaranteed or endorsed by the publisher.

## Footnotes

^1^ www.oceanario.pt
