# Supplementary material for: Does Predation Exacerbate the Risk of Endosymbiont Loss in Heat Stressed Hermatypic Corals? Molecular Cues Provide Insights Into Species-Specific Health Outcomes in a Multi-Stressor Ocean
Source: Front Physiol. 2022 Mar 1;13:801672. doi: 10.3389/fphys.2022.801672 (PMC8922028; doi:10.3389/fphys.2022.801672)
Supplement: Supplementary file 1 [file Data_Sheet_1.PDF]

SUPPLEMENTAL MATERIAL TO:

Does predation exacerbate the risk of endosymbiont loss in heat stressed hermatypic corals? Molecular cues provide insights into species-specific health outcomes in a multi-stressor world

Carolina Madeira[1,2,3*¶], Marta Dias[3*¶], Ana Ferreira[4], Raúl Gouveia[4], Henrique Cabral[3,5], Mário S. Diniz[1,2], Catarina Vinagre[3,6]

[¶] These authors contributed equally to this work.

[1]i4HB – Associate Laboratory Institute for Health and Bioeconomy, NOVA School of Science and Technology, NOVA University Lisbon, 2819-516 Caparica, Portugal

[2]UCIBIO – Applied Molecular Biosciences Unit, NOVA School of Science and Technology, NOVA University of Lisbon, 2829-516 Caparica, Portugal

[3]MARE – Marine and Environmental Sciences Centre, Faculty of Sciences, University of Lisbon, 1749-016 Lisboa, Portugal

[4]Oceanário de Lisboa, Esplanada D. Carlos I - 1990-005 Lisboa, Portugal

[5]INRAE – National Research Institute for Agriculture, Food the Environment, UR EABX, 50 Avenue de Verdun, 33612 Cestas, France

[6]CCMAR - Centre of Marine Sciences, University of Algarve, 8005-139 Faro, Portugal

*Corresponding authors:

Carolina Madeira, email: scg.madeira@fct.unl.pt, orcid: 0000-0003-1632-634X

Marta Dias, email: maddias@fc.ul.pt, orcid: 0000-0003-0447-6009

Running title: Coral predation in warming oceans

Table SM1. Post-hoc Tukey HSD tests for differences between species (all seven species) in biomarker concentrations, from Table 2A in the main manuscript (corals that survived at 30 ºC) (significant if p-value<0.05, presented in bold): a) Hsp70 (between MS = 0.451, df = 112.00); b) Ub (between MS = 0.339, df = 112.00); c) TAC (between MS = 0.289, df = 112.00).

a) Hsp70

	Species	{1}	{2}	{3}	{4}	{5}	{6}	{7}
		-.5283	.39537	-.4913	.14794	.12113	.29465	-.1236
1	*Acropora tenuis*		**0.000704**	0.999998	**0.030315**	**0.043215**	**0.003440**	0.481579
2	*Echinopora lamellosa*	**0.000704**		**0.001247**	0.905859	0.854879	0.999174	0.191098
3	*Galaxea fascicularis*	0.999998	**0.001247**		0.049253	0.068724	**0.006132**	0.597169
4	*Montipora capricornis* BM	**0.030315**	0.905859	**0.049253**		1.000000	0.992949	0.860609
5	*Montipora capricornis* GM	**0.043215**	0.854879	**0.068724**	1.000000		0.982850	0.910292
6	*Psammocora contigua*	**0.003440**	0.999174	**0.006132**	0.992949	0.982850		0.440453
7	*Turbinaria reniformis*	0.481579	0.191098	0.597169	0.860609	0.910292	0.440453	

b) Ub

	Species	{1}	{2}	{3}	{4}	{5}	{6}	{7}
		-.4517	.40806	-.5758	.21088	.01274	-.1203	-.1606
1	*Acropora tenuis*		**0.000288**	0.993834	**0.008552**	0.161487	0.550994	0.694591
2	*Echinopora lamellosa*	**0.000288**		**0.000130**	0.935401	0.333235	0.071134	**0.039641**
3	*Galaxea fascicularis*	0.993834	**0.000130**		**0.000903**	**0.029191**	0.179139	0.275516
4	*Montipora capricornis* BM	**0.008552**	0.935401	**0.000903**		0.933954	0.552080	0.409949
5	*Montipora capricornis* GM	0.161487	0.333235	**0.029191**	0.933954		0.991010	0.964982
6	*Psammocora contigua*	0.550994	0.071134	0.179139	0.552080	0.991010		0.999991
7	*Turbinaria reniformis*	0.694591	**0.039641**	0.275516	0.409949	0.964982	0.999991	

c) TAC

Species	{1}	{2}	{3}	{4}	{5}	{6}	{7}
	-.2989	1.7313	-.3290	-.1088	-.6620	-.0147	-.0266

1	*Acropora tenuis*		**0.000122**	0.999998	0.921706	0.340947	0.637407	0.682680
2	*Echinopora lamellosa*	**0.000122**		**0.000122**	**0.000122**	**0.000122**	**0.000122**	**0.000122**
3	*Galaxea fascicularis*	0.999998	**0.000122**		0.853484	0.448295	0.519887	0.566473
4	*Montipora capricornis* BM	0.921706	**0.000122**	0.853484		**0.024935**	0.997962	0.999083
5	*Montipora capricornis* GM	0.340947	**0.000122**	0.448295	**0.024935**		**0.004353**	**0.005488**
6	*Psammocora contigua*	0.637407	**0.000122**	0.519887	0.997962	**0.004353**		1.000000
7	*Turbinaria reniformis*	0.682680	**0.000122**	0.566473	0.999083	**0.005488**	1.000000	

Table SM2. Post-hoc Tukey HSD tests for differences between interaction of species (all seven species) with predation (no lesion vs with lesion) in biomarker concentrations from Table 2A (corals that survived at 30 ºC) in the main manuscript, significant if p-value<0.05 (presented in bold): a) Hsp70 (between MS = 0.451, df = 112.00); b) Ub (between MS = 0.339, df = 112.00); c) TAC (between MS = 0.289, df = 112.00).

a) Hsp70	Species	Predation	{1}	{2}	{3}	{4}	{5}	{6}	{7}	{8}	{9}	{10}	{11}	{12}	{13}	{14}
			-.4667	-.5899	.89761	-.1069	-.3014	-.6812	.42984	-.1340	-.2783	.52052	-.2018	.79108	-.1657	-.0815
1	*Acropora tenuis*	no lesion		1.000000	**0.001233**	0.995174	0.999999	0.999982	0.160103	0.997764	0.999996	0.074715	0.999805	**0.004367**	0.999222	0.990860
2	*Acropora tenuis*	with lesion	1.000000		**0.000332**	0.940581	0.999508	1.000000	0.055306	0.961670	0.998868	**0.022392**	0.990200	**0.001017**	0.978681	0.914483
3	*Echinopora lamellosa*	no lesion	**0.001233**	**0.000332**		0.063803	**0.008540**	**0.000185**	0.953290	**0.049445**	**0.011055**	0.992483	**0.025127**	1.000000	**0.036274**	0.080340
4	*Echinopora lamellosa*	with lesion	0.995174	0.940581	0.063803		0.999994	0.815877	0.877677	1.000000	0.999999	0.707721	1.000000	0.158306	1.000000	1.000000
5	*Galaxea fascicularis*	no lesion	0.999999	0.999508	**0.008540**	0.999994		0.991960	0.463365	0.999999	1.000000	0.273536	1.000000	**0.027000**	1.000000	0.999976
6	*Galaxea fascicularis*	with lesion	0.999982	1.000000	**0.000185**	0.815877	0.991960		**0.022249**	0.861769	0.986307	**0.008278**	0.943785	**0.000381**	0.906054	0.766771
7	*Montipora capricornis* BM	no lesion	0.160103	0.055306	0.953290	0.877677	0.463365	**0.022249**		0.834632	0.517840	1.000000	0.698261	0.994986	0.775272	0.911127
8	*Montipora capricornis* BM	with lesion	0.997764	0.961670	**0.049445**	1.000000	0.999999	0.861769	0.834632		1.000000	0.645710	1.000000	0.127560	1.000000	1.000000
9	*Montipora capricornis* GM	no lesion	0.999996	0.998868	**0.011055**	0.999999	1.000000	0.986307	0.517840	1.000000		0.317063	1.000000	**0.034158**	1.000000	0.999994

10	*Montipora capricornis* GM	with lesion	**0.074715**	**0.022392**	0.992483	0.707721	0.273536	**0.008278**	1.000000	0.645710	0.317063		0.484212	0.999754	0.570208	0.762153
11	*Psammocora contigua*	no lesion	0.999805	0.990200	**0.025127**	1.000000	1.000000	0.943785	0.698261	1.000000	1.000000	0.484212		0.070970	1.000000	1.000000
12	*Psammocora contigua*	with lesion	**0.004367**	**0.001017**	1.000000	0.158306	**0.027000**	**0.000381**	0.994986	0.127560	**0.034158**	0.999754	0.070970		0.097681	0.191929
13	*Turbinaria reniformis*	no lesion	0.999222	0.978681	**0.036274**	1.000000	1.000000	0.906054	0.775272	1.000000	1.000000	0.570208	1.000000	0.097681		1.000000
14	*Turbinaria reniformis*	with lesion	0.990860	0.914483	0.080340	1.000000	0.999976	0.766771	0.911127	1.000000	0.999994	0.762153	1.000000	0.191929	1.000000	

b) Ub	Species	Predation	{1}	{2}	{3}	{4}	{5}	{6}	{7}	{8}	{9}	{10}	{11}	{12}	{13}	{14}
			-.4358	-.4677	.73245	.08367	-.5427	-.6090	.09128	.33047	-.4963	.52175	-.5932	.35263	-.4253	.10412
1	*Acropora tenuis*	no lesion		1.000000	**0.001493**	0.767684	1.000000	0.999992	0.749519	0.176120	1.000000	**0.023770**	0.999998	0.144440	1.000000	0.717645
2	*Acropora tenuis*	with lesion	1.000000		**0.000976**	0.688002	1.000000	0.999999	0.667893	0.131915	1.000000	**0.016084**	1.000000	0.106733	1.000000	0.633293
3	*Echinopora lamellosa*	no lesion	**0.001493**	0.000976		0.423706	**0.000385**	**0.000216**	0.443680	0.956258	**0.000665**	0.999925	**0.000241**	0.972110	**0.001722**	0.478052
4	*Echinopora lamellosa*	with lesion	0.767684	0.688002	0.423706		0.483452	0.316440	1.000000	0.999573	0.611161	0.917931	0.353217	0.998918	0.791754	1.000000
5	*Galaxea fascicularis*	no lesion	1.000000	1.000000	**0.000385**	0.483452		1.000000	0.462922	0.062114	1.000000	**0.006139**	1.000000	**0.048802**	1.000000	0.428901
6	*Galaxea fascicularis*	with lesion	0.999992	0.999999	**0.000216**	0.316440	1.000000		0.299471	**0.029489**	1.000000	**0.002489**	1.000000	**0.022637**	0.999985	0.272088
7	*Montipora capricornis* BM	no lesion	0.749519	0.667893	0.443680	1.000000	0.462922	0.299471		0.999695	0.590204	0.927420	0.335184	0.999206	0.774417	1.000000
8	*Montipora capricornis* BM	with lesion	0.176120	0.131915	0.956258	0.999573	**0.062114**	**0.029489**	0.999695		0.100181	0.999975	**0.035434**	1.000000	0.192817	0.999833
9	*Montipora capricornis* GM	no lesion	1.000000	1.000000	**0.000665**	0.611161	1.000000	1.000000	0.590204	0.100181		**0.011221**	1.000000	**0.080114**	1.000000	0.554696
10	*Montipora capricornis* GM	with lesion	**0.023770**	**0.016084**	0.999925	0.917931	**0.006139**	**0.002489**	0.927420	0.999975	**0.011221**		**0.003126**	0.999994	**0.026955**	0.941691
11	*Psammocora contigua*	no lesion	0.999998	1.000000	**0.000241**	0.353217	1.000000	1.000000	0.335184	**0.035434**	1.000000	**0.003126**		**0.027341**	0.999995	0.305905
12	*Psammocora contigua*	with lesion	0.144440	0.106733	0.972110	0.998918	**0.048802**	**0.022637**	0.999206	1.000000	**0.080114**	0.999994	**0.027341**		0.158859	0.999540
13	*Turbinaria reniformis*	no lesion	1.000000	1.000000	**0.001722**	0.791754	1.000000	0.999985	0.774417	0.192817	1.000000	**0.026955**	0.999995	0.158859		0.743805
14	*Turbinaria reniformis*	with lesion	0.717645	0.633293	0.478052	1.000000	0.428901	0.272088	1.000000	0.999833	0.554696	0.941691	0.305905	0.999540	0.743805	

c) TAC	Species	Predation	{1}	{2}	{3}	{4}	{5}	{6}	{7}	{8}	{9}	{10}	{11}	{12}	{13}	{14}
			-.2927	-.3052	1.9627	1.4998	-.2485	-.4095	-.0293	-.1882	-.4071	-.9170	-.0462	.01685	-.3651	.31188
1	Acropora tenuis	no lesion		1.000000	**0.000134**	**0.000134**	1.000000	1.000000	0.998042	1.000000	1.000000	0.356872	0.999009	0.990594	1.000000	0.410025

2	Acropora tenuis	with lesion	1.000000		**0.000134**	**0.000134**	1.000000	1.000000	0.996863	1.000000	1.000000	0.390100	0.998361	0.986618	1.000000	0.376043
3	Echinopora lamellosa	no lesion	**0.000134**	**0.000134**		0.809695	**0.000134**	**0.000134**	**0.000134**	**0.000134**	**0.000134**	**0.000134**	**0.000134**	**0.000134**	**0.000134**	**0.000134**
4	Echinopora lamellosa	with lesion	**0.000134**	**0.000134**	0.809695		**0.000134**	**0.000134**	**0.000134**	**0.000134**	**0.000134**	**0.000134**	**0.000134**	**0.000135**	**0.000134**	**0.000347**
5	Galaxea fascicularis	no lesion	1.000000	1.000000	**0.000134**	**0.000134**		0.999992	0.999723	1.000000	0.999993	0.251724	0.999885	0.997875	1.000000	0.538492
6	Galaxea fascicularis	with lesion	1.000000	1.000000	**0.000134**	**0.000134**	0.999992		0.947930	0.999692	1.000000	0.694305	0.963301	0.884101	1.000000	0.155279
7	Montipora capricornis BM	no lesion	0.998042	0.996863	**0.000134**	**0.000134**	0.999723	0.947930		0.999993	0.950417	**0.022993**	1.000000	1.000000	0.980793	0.977974
8	Montipora capricornis BM	with lesion	1.000000	1.000000	**0.000134**	**0.000134**	1.000000	0.999692	0.999993		0.999727	0.144457	0.999998	0.999866	0.999975	0.714760
9	Montipora capricornis GM	no lesion	1.000000	1.000000	**0.000134**	**0.000134**	0.999993	1.000000	0.950417	0.999727		0.687352	0.965223	0.888362	1.000000	0.159039
10	Montipora capricornis GM	with lesion	0.356872	0.390100	**0.000134**	**0.000134**	0.251724	0.694305	**0.022993**	0.144457	0.687352		**0.028608**	**0.012394**	0.563807	**0.000235**
11	Psammocora contigua	no lesion	0.999009	0.998361	**0.000134**	**0.000134**	0.999885	0.963301	1.000000	0.999998	0.965223	**0.028608**		1.000000	0.987741	0.967273
12	Psammocora contigua	with lesion	0.990594	0.986618	**0.000134**	**0.000135**	0.997875	0.884101	1.000000	0.999866	0.888362	**0.012394**	1.000000		0.946157	0.993992
13	Turbinaria reniformis	no lesion	1.000000	1.000000	**0.000134**	**0.000134**	1.000000	1.000000	0.980793	0.999975	1.000000	0.563807	0.987741	0.946157		0.234112
14	Turbinaria reniformis	with lesion	0.410025	0.376043	**0.000134**	**0.000347**	0.538492	0.155279	0.977974	0.714760	0.159039	**0.000235**	0.967273	0.993992	0.234112	

Table SM3. Post-hoc Tukey HSD tests for differences between interaction of species (all seven species) with temperature (26 ºC vs 30 ºC) in biomarker concentrations from Table 2A (corals that survived at 30 ºC) in the main manuscript, significant if p-value<0.05 (presented in bold): a) Hsp70 (between MS = 0.451, df = 112.00); b) Ub (between MS = 0.339, df = 112.00); c) TAC (between MS = 0.289, df = 112.00).

a) Hsp70	Species	Temperature	{1}	{2}	{3}	{4}	{5}	{6}	{7}	{8}	{9}	{10}	{11}	{12}	{13}	{14}
			-.5677	-.4889	-.2473	1.0381	-.5333	-.4493	.46007	-.1642	.14568	.09658	-.5714	1.1607	-.3993	.15213
1	*Acropora tenuis*	26		1.000000	0.998499	**0.000168**	1.000000	1.000000	0.051254	0.986129	0.505230	0.622529	1.000000	**0.000139**	0.999999	0.489963
2	*Acropora tenuis*	30	1.000000		0.999930	**0.000246**	1.000000	1.000000	0.104536	0.998273	0.691694	0.795141	1.000000	**0.000151**	1.000000	0.677004
3	*Echinopora lamellosa*	26	0.998499	0.999930		**0.003159**	0.999554	0.999991	0.519491	1.000000	0.989030	0.996896	0.998317	**0.000739**	1.000000	0.987315
4	*Echinopora lamellosa*	30	**0.000168**	**0.000246**	**0.003159**		**0.000191**	**0.000333**	0.809134	**0.008229**	0.165223	0.111281	**0.000166**	1.000000	**0.000538**	0.173572
5	*Galaxea fascicularis*	26	1.000000	1.000000	0.999554	**0.000191**		1.000000	0.070671	0.993860	0.587679	0.702248	1.000000	**0.000142**	1.000000	0.572227

6	*Galaxea fascicularis*	30	1.000000	1.000000	0.999991	**0.000333**	1.000000		0.144666	0.999566	0.776329	0.863869	1.000000	**0.000166**	1.000000	0.763246
7	*Montipora capricornis* BM	26	0.051254	0.104536	0.519491	0.809134	0.070671	0.144666		0.714679	0.998762	0.994683	**0.049507**	0.535749	0.211282	0.999000
8	*Montipora capricornis* BM	30	0.986129	0.998273	1.000000	**0.008229**	0.993860	0.999566	0.714679		0.998934	0.999836	0.984991	**0.001957**	0.999949	0.998682
9	*Montipora capricornis* GM	26	0.505230	0.691694	0.989030	0.165223	0.587679	0.776329	0.998762	0.998934		1.000000	0.496616	0.057845	0.865307	1.000000
10	*Montipora capricornis* GM	30	0.622529	0.795141	0.996896	0.111281	0.702248	0.863869	0.994683	0.999836	1.000000		0.613903	**0.035996**	0.928200	1.000000
11	*Psammocora contigua*	26	1.000000	1.000000	0.998317	**0.000166**	1.000000	1.000000	**0.049507**	0.984991	0.496616	0.613903		**0.000138**	0.999999	0.481409
12	*Psammocora contigua*	30	**0.000139**	0.000151	**0.000739**	1.000000	**0.000142**	**0.000166**	0.535749	**0.001957**	0.057845	**0.035996**	**0.000138**		**0.000202**	0.061442
13	*Turbinaria reniformis*	26	0.999999	1.000000	1.000000	**0.000538**	1.000000	1.000000	0.211282	0.999949	0.865307	0.928200	0.999999	**0.000202**		0.855187
14	*Turbinaria reniformis*	30	0.489963	0.677004	0.987315	0.173572	0.572227	0.763246	0.999000	0.998682	1.000000	1.000000	0.481409	0.061442	0.855187	

b) Ub	Species	Temperature	{1}	{2}	{3}	{4}	{5}	{6}	{7}	{8}	{9}	{10}	{11}	{12}	{13}	{14}
			-.6048	-.2987	-.2461	1.0623	-.7662	-.3854	.46110	-.0393	.01541	.01007	-1.162	.92163	-.6294	.30828
1	*Acropora tenuis*	26		0.995969	0.982745	**0.000134**	0.999997	0.999882	**0.006021**	0.650615	0.500153	0.514772	0.672154	**0.000137**	1.000000	**0.039987**
2	*Acropora tenuis*	30	0.995969		1.000000	**0.000192**	0.873816	1.000000	0.186243	0.999278	0.994832	0.995618	0.068813	**0.000740**	0.991619	0.536497
3	*Echinopora lamellosa*	26	0.982745	1.000000		**0.000278**	0.766305	0.999999	0.284339	0.999939	0.999200	0.999365	**0.038657**	**0.001502**	0.969969	0.680039
4	*Echinopora lamellosa*	30	**0.000134**	**0.000192**	**0.000278**		**0.000134**	**0.000146**	0.552572	**0.003744**	**0.007729**	**0.007209**	**0.000134**	0.999999	**0.000134**	0.195735
5	*Galaxea fascicularis*	26	0.999997	0.873816	0.766305	**0.000134**		0.971563	**0.000677**	0.244568	0.153694	0.161242	0.961055	**0.000134**	1.000000	**0.005378**
6	*Galaxea fascicularis*	30	0.999882	1.000000	0.999999	**0.000146**	0.971563		0.082071	0.987360	0.957187	0.961441	0.160518	**0.000281**	0.999622	0.313948
7	*Montipora capricornis BM*	26	**0.006021**	0.186243	0.284339	0.552572	**0.000677**	0.082071		0.810343	0.907657	0.899974	**0.000134**	0.885318	**0.004345**	0.999998
8	*Montipora capricornis BM*	30	0.650615	0.999278	0.999939	**0.003744**	0.244568	0.987360	0.810343		1.000000	1.000000	**0.002789**	**0.022805**	0.583201	0.986863
9	*Montipora capricornis GM*	26	0.500153	0.994832	0.999200	**0.007729**	0.153694	0.957187	0.907657	1.000000		1.000000	**0.001316**	**0.043201**	0.434004	0.997392
10	*Montipora capricornis GM*	30	0.514772	0.995618	0.999365	**0.007209**	0.161242	0.961441	0.899974	1.000000	1.000000		**0.001414**	**0.040681**	0.448092	0.996885
11	*Psammocora contigua*	26	0.672154	0.068813	**0.038657**	**0.000134**	0.961055	0.160518	**0.000134**	**0.002789**	**0.001316**	**0.001414**		**0.000134**	0.735525	**0.000142**
12	*Psammocora contigua*	30	**0.000137**	**0.000740**	**0.001502**	0.999999	**0.000134**	**0.000281**	0.885318	**0.022805**	**0.043201**	**0.040681**	**0.000134**		**0.000136**	0.518951
13	*Turbinaria reniformis*	26	1.000000	0.991619	0.969969	**0.000134**	1.000000	0.999622	**0.004345**	0.583201	0.434004	0.448092	0.735525	**0.000136**		**0.030088**
14	*Turbinaria reniformis*	30	**0.039987**	0.536497	0.680039	0.195735	**0.005378**	0.313948	0.999998	0.986863	0.997392	0.996885	**0.000142**	0.518951	**0.030088**	

c) TAC	Species	Temperature	{1}	{2}	{3}	{4}	{5}	{6}	{7}	{8}	{9}	{10}	{11}	{12}	{13}	{14}
			.03831	-.6362	1.0723	2.3902	.27622	-.9343	-.8492	.63158	-.9522	-.3719	.10269	-.1321	-.0005	-.0527
1	*Acropora tenuis*	26		0.239076	**0.002961**	**0.000134**	0.999334	**0.007210**	**0.023052**	0.442004	**0.005591**	0.910288	1.000000	0.999984	1.000000	1.000000
2	*Acropora tenuis*	30	0.239076		**0.000134**	**0.000134**	**0.016572**	0.993380	0.999797	**0.000182**	0.988691	0.997975	0.130515	0.703644	0.327579	0.470291
3	*Echinopora lamellosa*	26	**0.002961**	**0.000134**		**0.000152**	0.070517	**0.000134**	**0.000134**	0.857429	**0.000134**	**0.000135**	**0.007526**	**0.000292**	**0.001658**	**0.000772**
4	*Echinopora lamellosa*	30	**0.000134**	**0.000134**	**0.000152**		**0.000134**	**0.000134**	**0.000134**	**0.000134**	**0.000134**	**0.000134**	**0.000134**	**0.000134**	**0.000134**	**0.000134**
5	*Galaxea fascicularis*	26	0.999334	**0.016572**	**0.070517**	**0.000134**		**0.000275**	**0.000768**	0.969241	**0.000236**	0.297427	0.999980	0.913157	0.996766	0.983875
6	*Galaxea fascicularis*	30	**0.007210**	0.993380	**0.000134**	**0.000134**	**0.000275**		1.000000	**0.000134**	1.000000	0.532632	**0.002828**	**0.065707**	**0.012397**	**0.024890**
7	*Montipora capricornis* BM	26	**0.023052**	0.999797	**0.000134**	**0.000134**	**0.000768**	1.000000		**0.000135**	1.000000	0.775066	**0.009655**	0.161942	**0.037750**	**0.070209**
8	*Montipora capricornis* BM	30	0.442004	**0.000182**	0.857429	**0.000134**	0.969241	**0.000134**	**0.000135**		**0.000134**	**0.004639**	0.632446	0.100855	0.336910	0.219464
9	*Montipora capricornis* GM	26	**0.005591**	0.988691	**0.000134**	**0.000134**	**0.000236**	1.000000	1.000000	**0.000134**		0.479730	**0.002158**	0.053348	**0.009674**	**0.019697**
10	*Montipora capricornis* GM	30	0.910288	0.997975	**0.000135**	**0.000134**	0.297427	0.532632	0.775066	**0.004639**	0.479730		0.781588	0.999274	0.956354	0.987615
11	*Psammocora contigua*	26	1.000000	0.130515	**0.007526**	**0.000134**	0.999980	**0.002828**	**0.009655**	0.632446	**0.002158**	0.781588		0.999421	1.000000	0.999995
12	*Psammocora contigua*	30	0.999984	0.703644	**0.000292**	**0.000134**	0.913157	0.065707	0.161942	0.100855	0.053348	0.999274	0.999421		0.999999	1.000000
13	*Turbinaria reniformis*	26	1.000000	0.327579	**0.001658**	**0.000134**	0.996766	**0.012397**	**0.037750**	0.336910	**0.009674**	0.956354	1.000000	0.999999		1.000000
14	*Turbinaria reniformis*	30	1.000000	0.470291	**0.000772**	**0.000134**	0.983875	**0.024890**	**0.070209**	0.219464	**0.019697**	0.987615	0.999995	1.000000	1.000000	

Table SM4. Post-hoc Tukey HSD tests for differences between interaction of predation (no lesion vs with lesion) with temperature (26 ºC vs 30 ºC) in TAC concentrations (between MS = 0.289, df = 112.00) from Table 2A (corals that survived at 30 ºC) in the main manuscript, significant if p-value<0.05 (presented in bold).

TAC	Predation	Temperature	{1}	{2}	{3}	{4}
			-.1552	.31912	.06598	-.0635
1	no lesion	26		**0.002070**	0.318909	0.892043
2	no lesion	30	**0.002070**		0.206700	**0.018732**
3	with lesion	26	0.318909	0.206700		0.746099
4	with lesion	30	0.892043	**0.018732**	0.746099	

Note 1: Post-hoc Tukey HSD tests are not shown for 'Temperature' and 'Predation' factors alone from Table 2A, because they only contain two categories: 26 ºC vs 30 ºC and no lesion vs lesion, respectively. All significant differences found are therefore between the two categories of each factor.

Note 2: Given the complexity of interaction tables for factors Species × Predation × Temperature in Table 2A of the main manuscript, only a few interactions of interest were selected and are presented in Fig. SM1 to SM4 (see below).

Table SM5. Post-hoc Tukey HSD tests for differences between species (*G. fascicularis* vs *P. contigua* vs *T. reniformis*) in biomarker concentrations from Table 2B (corals that survived at 32 ºC) in the main manuscript, significant if p-value<0.05 (presented in bold): a) Hsp70 (between MS = 0.509, df = 72.000), b) Ub (between MS = 0.298, df = 72.000); and c) TAC (between MS = 0.324, df = 72.000).

a) Hsp70	Species	{1}	{2}	{3}
		-.5328	.58876	-.1467
1	*Galaxea fascicularis*		**0.000111**	0.098145
2	*Psammocora contigua*	**0.000111**		**0.000550**
3	*Turbinaria reniformis*	0.098145	**0.000550**	

b) Ub	Species	{1}	{2}	{3}
		-.5270	.53468	-.1277
1	*Galaxea fascicularis*		**0.000111**	**0.016524**
2	*Psammocora contigua*	**0.000111**		**0.000143**
3	*Turbinaria reniformis*	**0.016524**	**0.000143**	

c) TAC	Species	{1}	{2}	{3}
		-.3990	-.0735	.03155
1	*Galaxea fascicularis*		0.075517	**0.012568**
2	*Psammocora contigua*	0.075517		0.755669
3	*Turbinaria reniformis*	**0.012568**	0.755669	

Table SM6. Post-hoc Tukey HSD tests for differences between temperature (26 ºC vs 30 ºC vs 32 ºC) in biomarker concentrations from Table 2B (corals that survived at 32 ºC) in the main manuscript, significant if p-value<0.05 (presented in bold): a) Hsp70 (between MS = 0.509, df = 72.000), b) Ub (between MS = 0.298, df = 72.000); and c) TAC (between MS = 0.324, df = 72.000).

a) Hsp70	Temperature	{1}	{2}	{3}
		-.5013	.28782	.12272
1	26		**0.000265**	**0.003366**
2	30	**0.000265**		0.644844
3	32	**0.003366**	0.644844	

b) Ub	Temperature	{1}	{2}	{3}
		-.8526	.28149	.45116

1	26		**0.000111**	**0.000111**
2	30	**0.000111**		0.455668
3	32	**0.000111**	0.455668	

c) TAC	Temperature	{1}	{2}	{3}
		.12615	-.3730	-.1941
1	26		**0.003256**	0.081768
2	30	**0.003256**		0.446923
3	32	0.081768	0.446923	

Table SM7. Post-hoc Tukey HSD tests for differences between interaction of species (*G. fascicularis* vs *P. contigua* vs *T. reniformis*) and predation (no lesion vs with lesion) in biomarker concentrations from Table 2B (corals that survived at 32 ºC) in the main manuscript, significant if p-value<0.05 (presented in bold): a) Hsp70 (between MS = 0.509, df = 72.000), b) Ub (between MS = 0.298, df = 72.000); and c) TAC (between MS = 0.324, df = 72.000).

a) Hsp70	Species	Predation	{1}	{2}	{3}	{4}	{5}	{6}
			-.4092	-.6564	-.0696	1.2472	-.3167	.02328
1	*Galaxea fascicularis*	no lesion		0.932416	0.782660	**0.000128**	0.999275	0.562889
2	*Galaxea fascicularis*	with lesion	0.932416		0.228100	**0.000127**	0.782384	0.108753
3	*Psammocora contigua*	no lesion	0.782660	0.228100		**0.000166**	0.932561	0.999260
4	*Psammocora contigua*	with lesion	**0.000128**	**0.000127**	**0.000166**		**0.000128**	**0.000294**
5	*Turbinaria reniformis*	no lesion	0.999275	0.782384	0.932561	**0.000128**		0.781862
6	*Turbinaria reniformis*	with lesion	0.562889	0.108753	0.999260	**0.000294**	0.781862	

b) Ub	Species	Predation	{1}	{2}	{3}	{4}	{5}	{6}

			-.5879	-.4661	-.1753	1.2447	-.4810	.22565
1	*Galaxea fascicularis*	no lesion		0.990034	0.315943	**0.000127**	0.994570	**0.001681**
2	*Galaxea fascicularis*	with lesion	0.990034		0.692119	**0.000127**	1.000000	**0.011201**
3	*Psammocora contigua*	no lesion	0.315943	0.692119		**0.000127**	0.645225	0.347502
4	*Psammocora contigua*	with lesion	**0.000127**	**0.000127**	**0.000127**		**0.000127**	**0.000158**
5	*Turbinaria reniformis*	no lesion	0.994570	1.000000	0.645225	**0.000127**		**0.008964**
6	*Turbinaria reniformis*	with lesion	**0.001681**	**0.011201**	0.347502	**0.000158**	0.008964	

c) TAC	Species	Predation	{1}	{2}	{3}	{4}	{5}	{6}
			-.1436	-.6545	-.1033	-.0437	-.1883	.25141
1	*Galaxea fascicularis*	no lesion		0.150715	0.999965	0.996759	0.999940	0.410525
2	*Galaxea fascicularis*	with lesion	0.150715		0.098241	**0.048701**	0.231542	**0.000704**
3	*Psammocora contigua*	no lesion	0.999965	0.098241		0.999753	0.998535	0.532050
4	*Psammocora contigua*	with lesion	0.996759	**0.048701**	0.999753		0.981916	0.715112
5	*Turbinaria reniformis*	no lesion	0.999940	0.231542	0.998535	0.981916		0.291192
6	*Turbinaria reniformis*	with lesion	0.410525	**0.000704**	0.532050	0.715112	0.291192	

Table SM8. Post-hoc Tukey HSD tests for differences between interaction of species (*G. fascicularis* vs *P. contigua* vs *T. reniformis*) and temperature (26 ºC vs 30 ºC vs 32 ºC) in biomarker concentrations from Table 2B (corals that survived at 32 ºC) in the main manuscript, significant if p-value<0.05 (presented in bold): a) Hsp70 (between MS = 0.509, df = 72.000), b) Ub (between MS = 0.298, df = 72.000); and c) TAC (between MS = 0.324, df = 72.000).

a) Hsp70	Species	Temperature	{1}	{2}	{3}	{4}	{5}	{6}	{7}	{8}	{9}
			-.5333	-.4493	-.6159	-.5714	1.1607	1.1770	-.3993	.15213	-.1929
1	*Galaxea fascicularis*	26		0.999999	0.999999	1.000000	**0.000169**	**0.000163**	0.999972	0.451006	0.977295
2	*Galaxea fascicularis*	30	0.999999		0.999855	0.999986	**0.000233**	**0.000215**	1.000000	0.626811	0.996485

3	*Galaxea fascicularis*	32	0.999999	0.999855		1.000000	**0.000147**	**0.000145**	0.998962	0.297066	0.920593
4	*Psammocora contigua*	26	1.000000	0.999986	1.000000		**0.000156**	**0.000152**	0.999815	0.376245	0.957163
5	*Psammocora contigua*	30	**0.000169**	**0.000233**	**0.000147**	**0.000156**		1.000000	**0.000316**	0.055524	**0.002130**
6	*Psammocora contigua*	32	**0.000163**	**0.000215**	**0.000145**	**0.000152**	1.000000		**0.000283**	0.048511	**0.001807**
7	*Turbinaria reniformis*	26	0.999972	1.000000	0.998962	0.999815	**0.000316**	0.000283		0.728025	0.999290
8	*Turbinaria reniformis*	30	0.451006	0.626811	0.297066	0.376245	0.055524	0.048511	0.728025		0.975289
9	*Turbinaria reniformis*	32	0.977295	0.996485	0.920593	0.957163	**0.002130**	**0.001807**	0.999290	0.975289	

b) Ub	Species	Temperature	{1}	{2}	{3}	{4}	{5}	{6}	{7}	{8}	{9}
			-.7662	-.3854	-.4293	-1.162	.92163	1.8446	-.6294	.30828	-.0618
1	*Galaxea fascicularis*	26		0.823848	0.902355	0.790576	**0.000136**	**0.000136**	0.999755	**0.001285**	0.110571
2	*Galaxea fascicularis*	30	0.823848		1.000000	0.052768	**0.000164**	**0.000136**	0.984948	0.122303	0.920771
3	*Galaxea fascicularis*	32	0.902355	1.000000		0.083513	**0.000149**	**0.000136**	0.995984	0.079566	0.850251
4	*Psammocora contigua*	26	0.790576	0.052768	0.083513		**0.000136**	**0.000136**	0.429677	**0.000138**	**0.000918**
5	*Psammocora contigua*	30	**0.000136**	**0.000164**	**0.000149**	**0.000136**		**0.009369**	**0.000137**	0.245021	**0.004276**
6	*Psammocora contigua*	32	**0.000136**	**0.000136**	**0.000136**	**0.000136**	**0.009369**		**0.000136**	**0.000137**	**0.000136**
7	*Turbinaria reniformis*	26	0.999755	0.984948	0.995984	0.429677	**0.000137**	**0.000136**		**0.007767**	0.342963
8	*Turbinaria reniformis*	30	**0.001285**	0.122303	0.079566	**0.000138**	0.245021	**0.000137**	**0.007767**		0.845231
9	*Turbinaria reniformis*	32	0.110571	0.920771	0.850251	**0.000918**	**0.004276**	**0.000136**	0.342963	0.845231	

c) TAC	Species	Temperature	{1}	{2}	{3}	{4}	{5}	{6}	{7}	{8}	{9}
			.27622	-.9343	-.5390	.10269	-.1321	-.1911	-.0005	-.0527	.14783
1	*Galaxea fascicularis*	26		**0.000435**	**0.049476**	0.998925	0.799632	0.658652	0.974427	0.930504	0.999887
2	*Galaxea fascicularis*	30	**0.000435**		0.826401	**0.003644**	0.056620	0.101199	**0.013075**	**0.024026**	**0.002054**
3	*Galaxea fascicularis*	32	**0.049476**	0.826401		0.239792	0.802461	0.906515	0.471269	0.609079	0.167054
4	*Psammocora contigua*	26	0.998925	**0.003644**	0.239792		0.991006	0.963317	0.999979	0.999532	1.000000
5	*Psammocora contigua*	30	0.799632	0.056620	0.802461	0.991006		1.000000	0.999864	0.999997	0.972558

6	*Psammocora contigua*	32	0.658652	0.101199	0.906515	0.963317	1.000000		0.997849	0.999802	0.918425
7	*Turbinaria reniformis*	26	0.974427	**0.013075**	0.471269	0.999979	0.999864	0.997849		1.000000	0.999669
8	*Turbinaria reniformis*	30	0.930504	**0.024026**	0.609079	0.999532	0.999997	0.999802	1.000000		0.996938
9	*Turbinaria reniformis*	32	0.999887	**0.002054**	0.167054	1.000000	0.972558	0.918425	0.999669	0.996938	

Table SM9. Post-hoc Tukey HSD tests for differences between interaction of predation (no lesion vs with lesion) and temperature (26 ºC vs 30 ºC vs 32 ºC) in ubiquitin concentrations (between MS = 0.298, df = 72.000) from Table 2B (corals that survived at 32 ºC) in the main manuscript, significant if p-value<0.05 (presented in red).

Ub	Predation	Temperature	{1}	{2}	{3}	{4}	{5}	{6}
			-1.014	-.0268	-.2034	-.6913	.58979	1.1057
1	no lesion	26		**0.000187**	**0.001760**	0.590265	**0.000127**	**0.000127**
2	no lesion	30	**0.000187**		0.949052	**0.016719**	**0.032659**	**0.000130**
3	no lesion	32	**0.001760**	0.949052		0.154976	**0.002315**	**0.000127**
4	with lesion	26	0.590265	**0.016719**	0.154976		**0.000127**	**0.000127**
5	with lesion	30	**0.000127**	**0.032659**	**0.002315**	**0.000127**		0.114296
6	with lesion	32	**0.000127**	**0.000130**	**0.000127**	**0.000127**	0.114296	

Note 3: Post-hoc Tukey HSD tests are not shown for 'Predation' factor from Table 2B, because it only contains two categories: no lesion vs lesion. All significant differences found are therefore between these two categories.

Note 4: Given the complexity of interaction tables for factor interactions Species × Predation × Temperature in Table 2B, only a few interactions of interest were selected and are presented in Fig. SM5 to SM7 (see below).

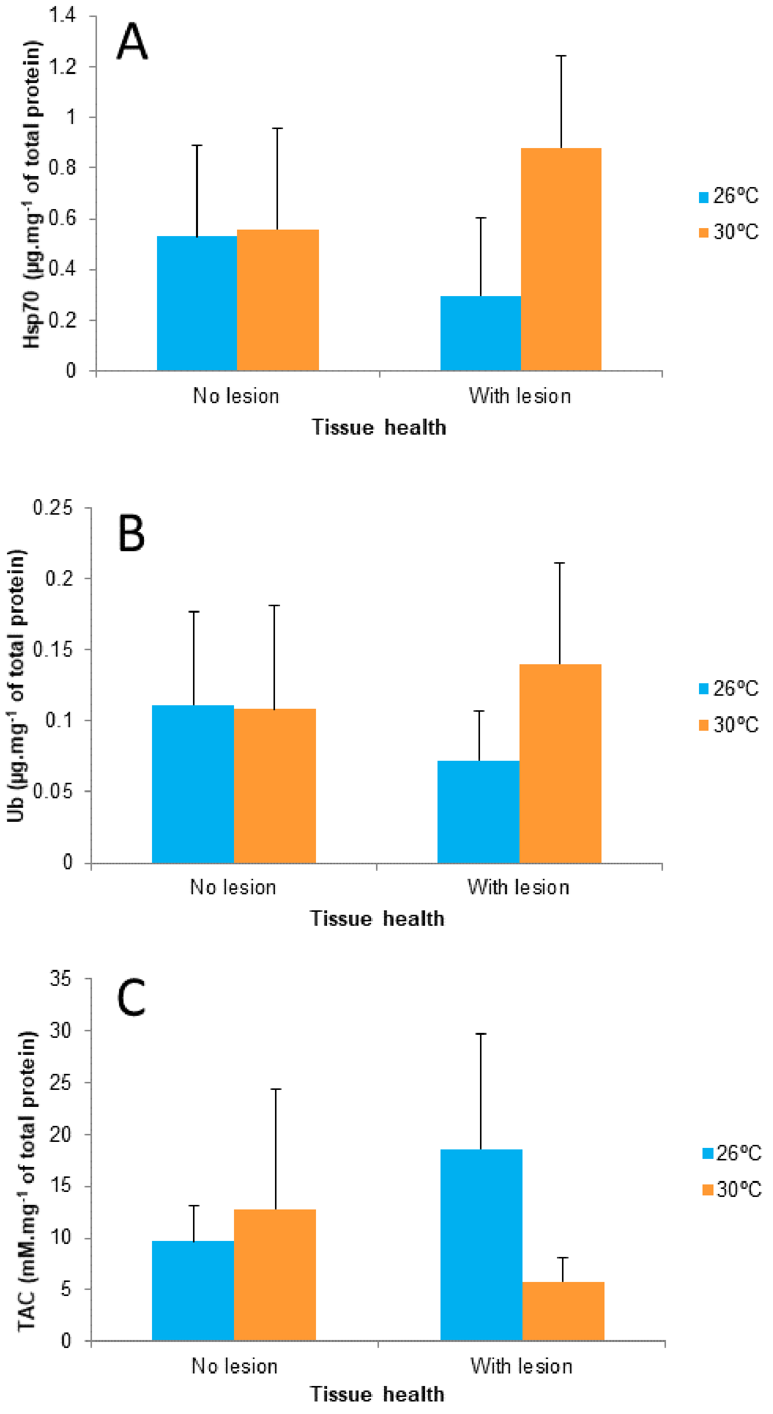

Fig. SM1 Biomarker values measured in the coral species *Acropora tenuis* (selected interactive effects between species × temperature × predation from Table 2A). (A) Heat shock protein 70 kDa, (B) Total ubiquitin, (C) Total antioxidant capacity.

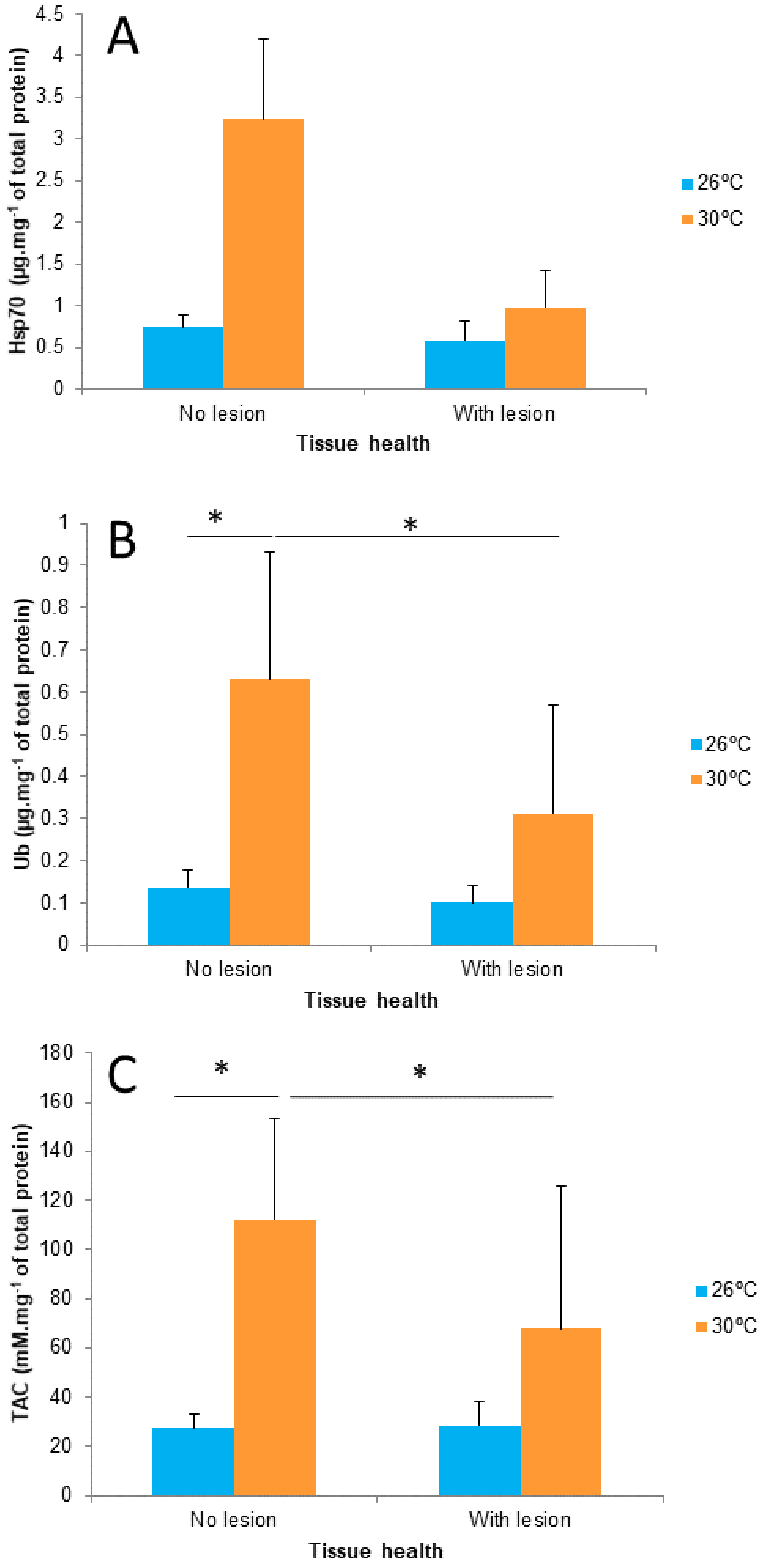

Fig. SM2 Biomarker values measured in the coral species *Echinopora lamellosa* (selected interactive effects between species × temperature × predation from Table 2A). (A) Heat shock protein 70 kDa, (B) Total ubiquitin, (C) Total antioxidant capacity.

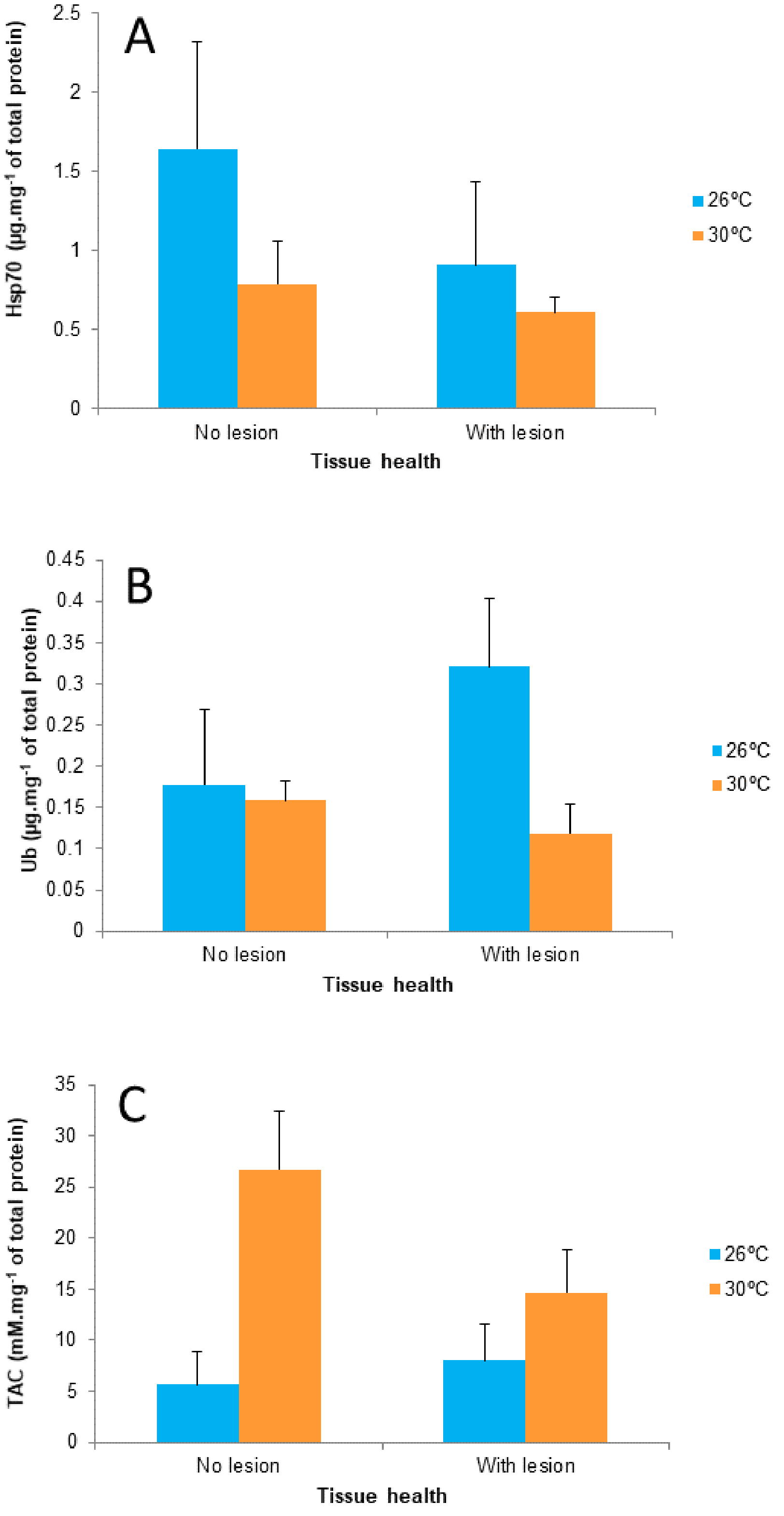

Fig. SM3 Biomarker values measured in the coral species *Montipora capricornis* brown morphotype (selected interactive effects between species × temperature × predation from Table 2A). (A) Heat shock protein 70 kDa, (B) Total ubiquitin, (C) Total antioxidant capacity.

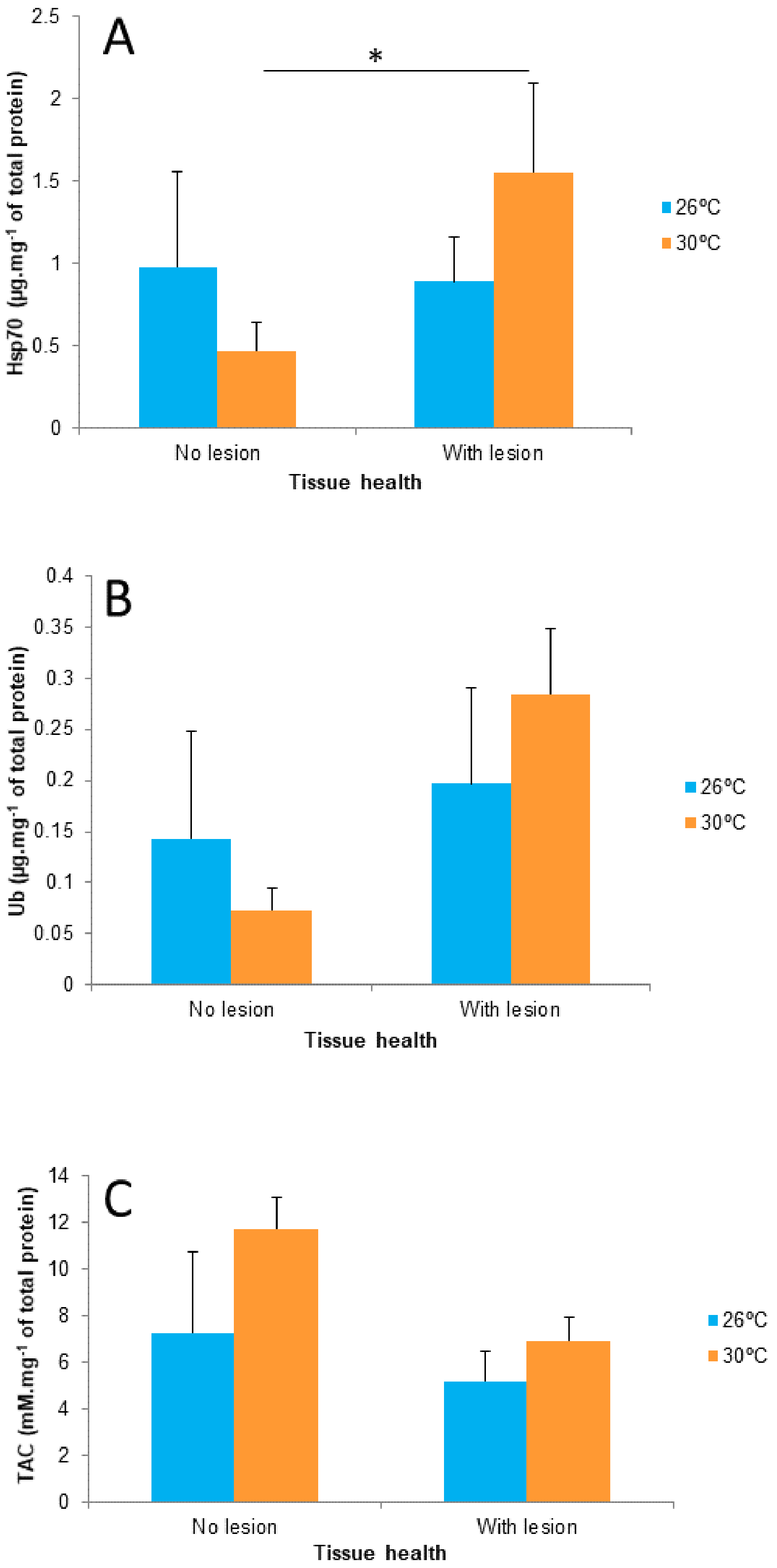

Fig. SM4 Biomarker values measured in the coral species *Montipora capricornis* green morphotype (selected interactive effects between species × temperature × predation from Table 2A). (A) Heat shock protein 70 kDa, (B) Total ubiquitin, (C) Total antioxidant capacity.

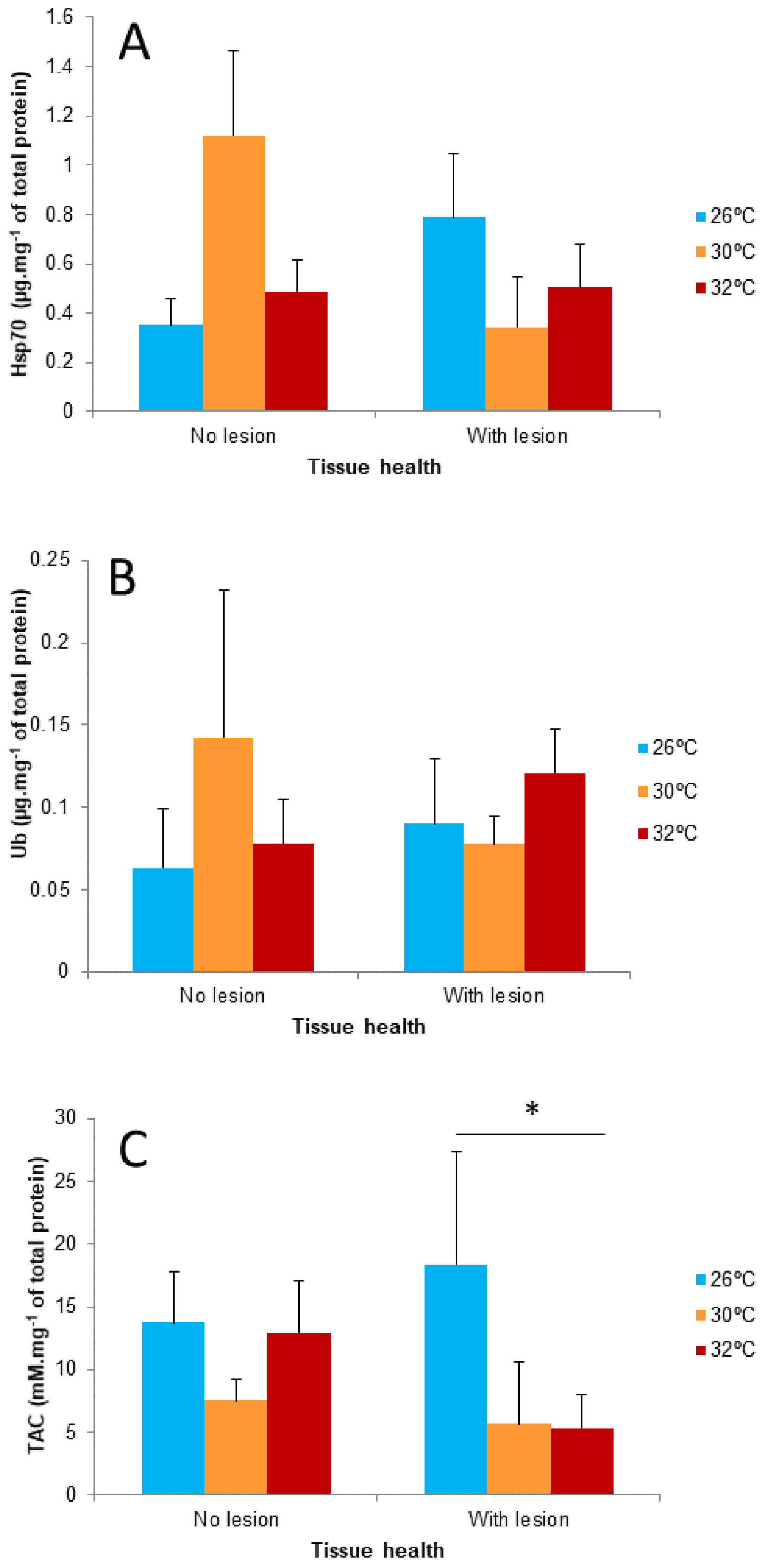

Fig. SM5 Biomarker values measured in the coral species *Galaxea fascicularis* (selected interactive effects between species × temperature × predation from Table 2B). (A) Heat shock protein 70 kDa, (B) Total ubiquitin, (C) Total antioxidant capacity.

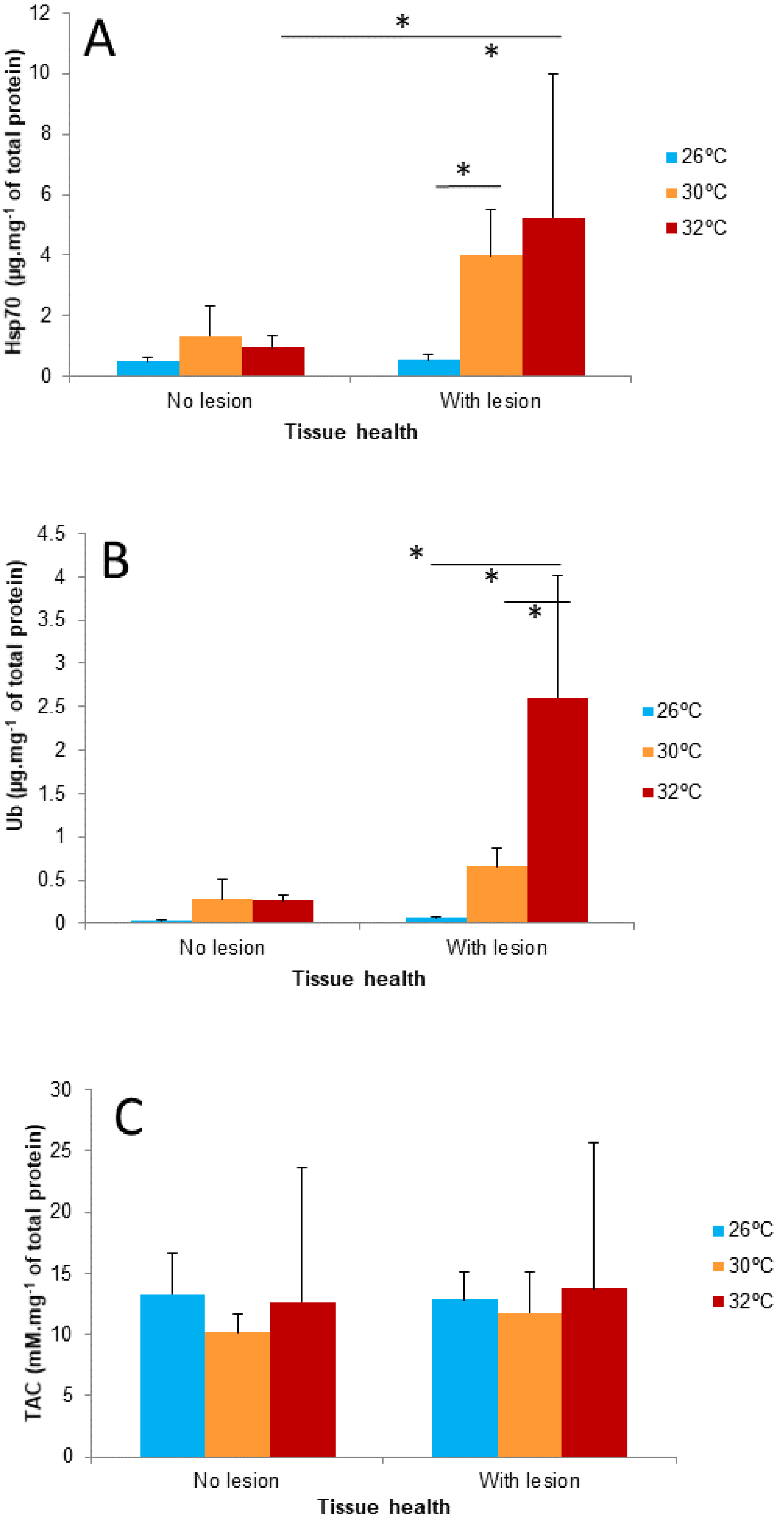

Fig. SM6 Biomarker values measured in the coral species *Psammocora contigua* (selected interactive effects between species × temperature × predation from Table 2B). (A) Heat shock protein 70 kDa, (B) Total ubiquitin, (C) Total antioxidant capacity.

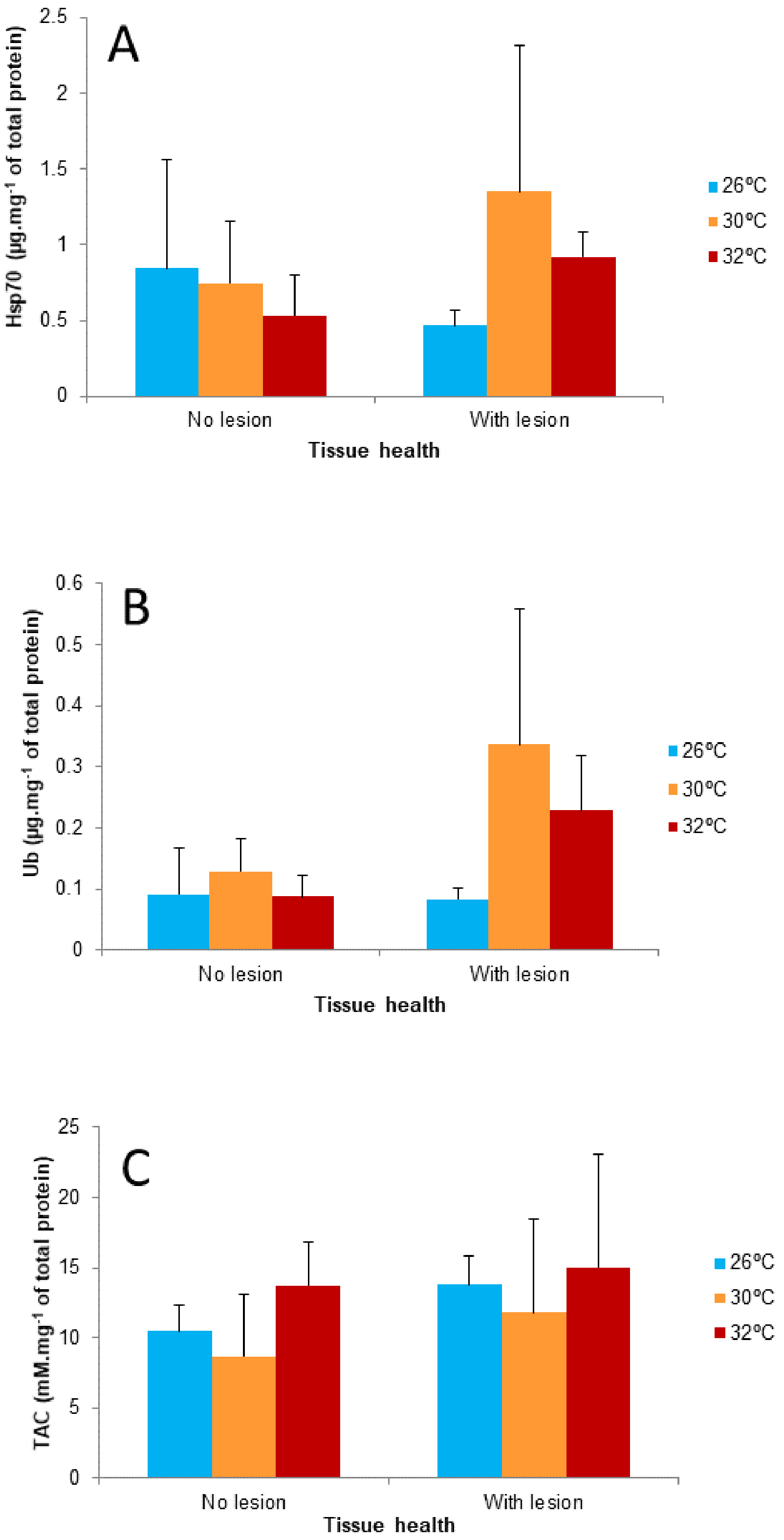

Fig. SM7 Biomarker values measured in the coral species *Turbinaria reniformis* (selected interactive effects between species × temperature × predation from Table 2B). (A) Heat shock protein 70 kDa, (B) Total ubiquitin, (C) Total antioxidant capacity.

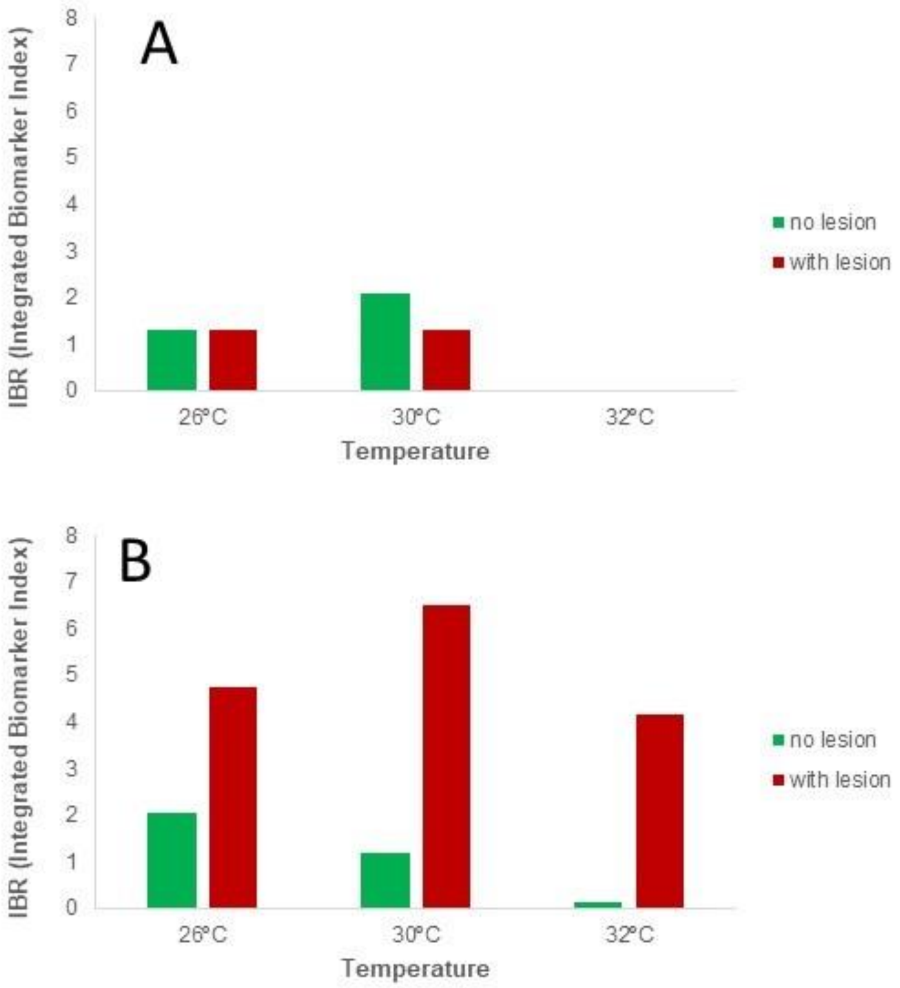

Fig. SM8 IBR values calculated for branching species: A) *Acropora tenuis* and B) *Psammocora contigua*. Note: the absence of values at 32 ºC means that the species died at that temperature.

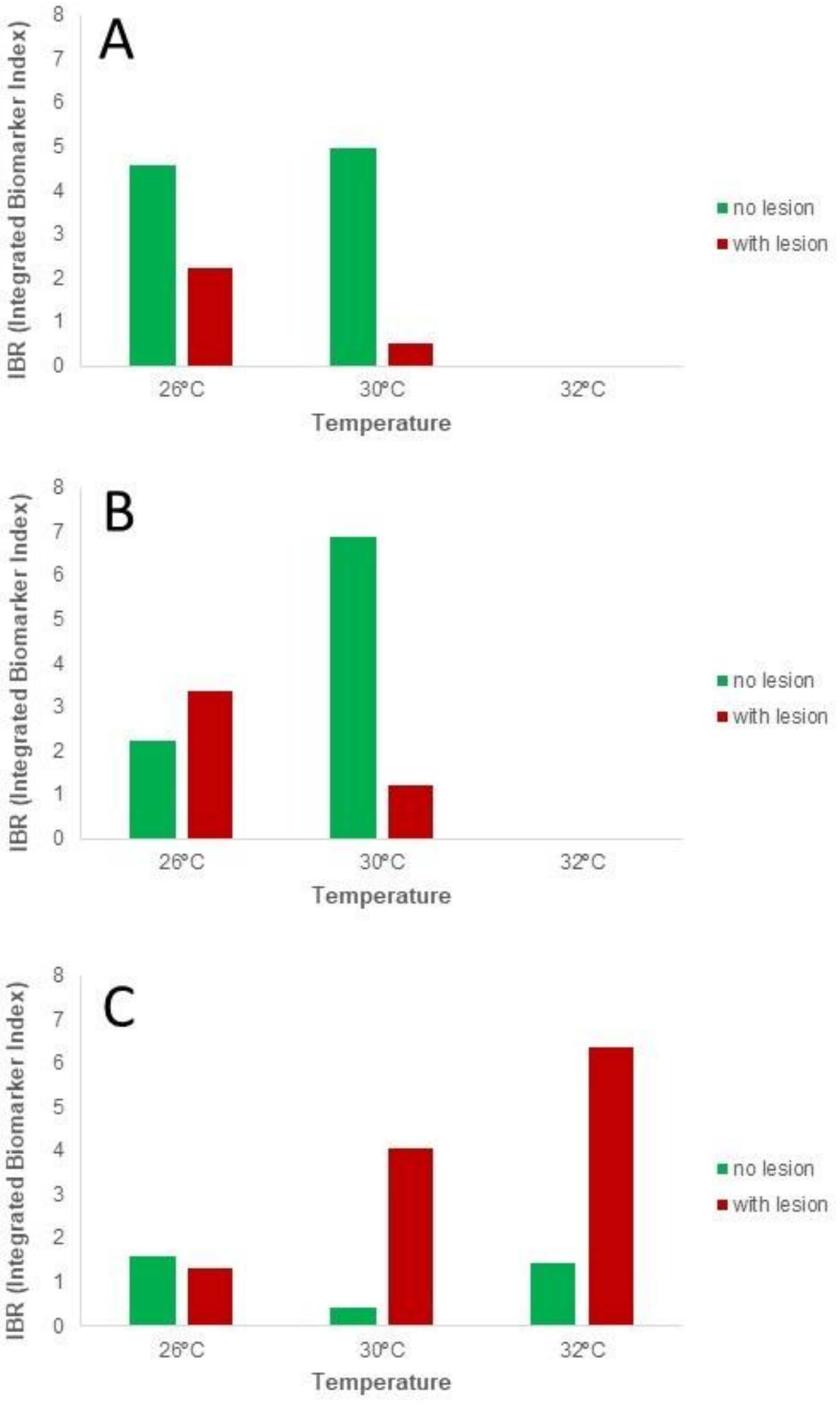

Fig. SM9 IBR values calculated for plating species: A) *Echinopora lamellosa*, B) *Montipora capricornis* BM and C) *Turbinaria reniformis*. Note: the absence of values at 32 ºC means that the species died at that temperature.

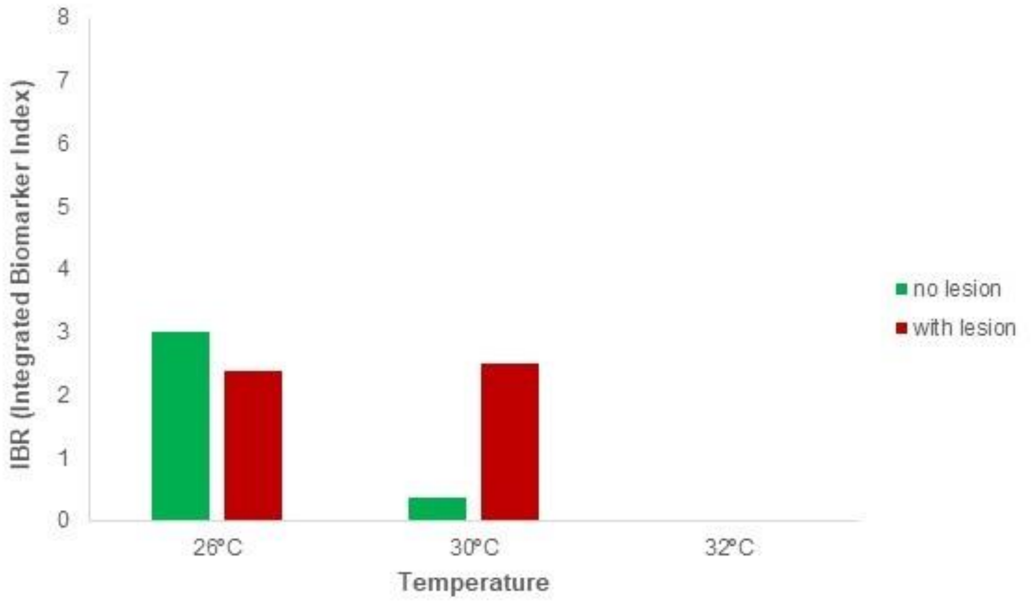

Fig. SM10 IBR values calculated for encrusting species *Montipora capricornis* GM. Note: the absence of values at 32 ºC means that the species died at that temperature.

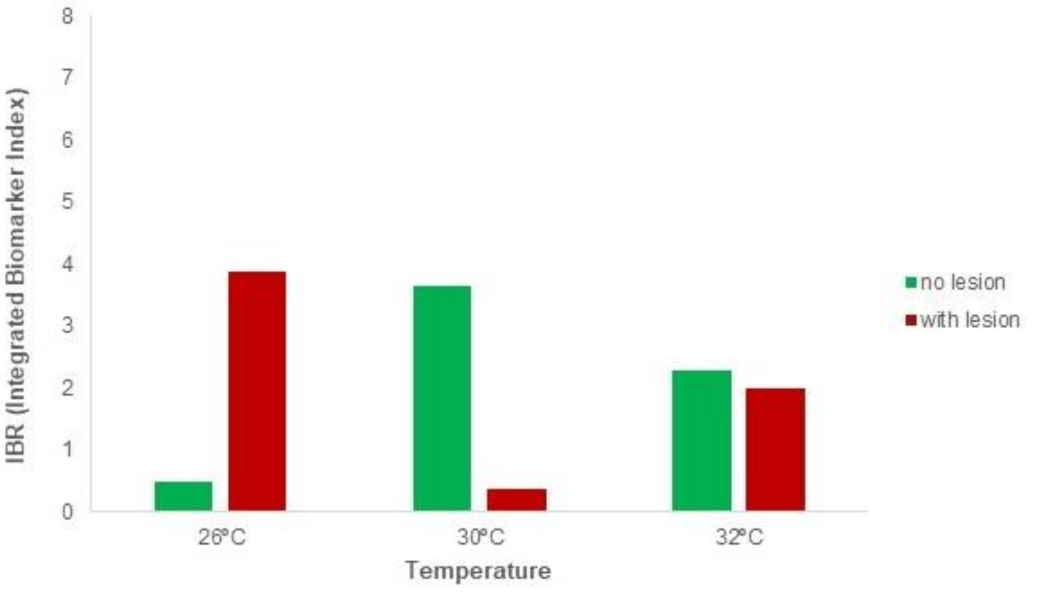

Fig. SM11 IBR values calculated for massive species *Galaxea fascicularis*.

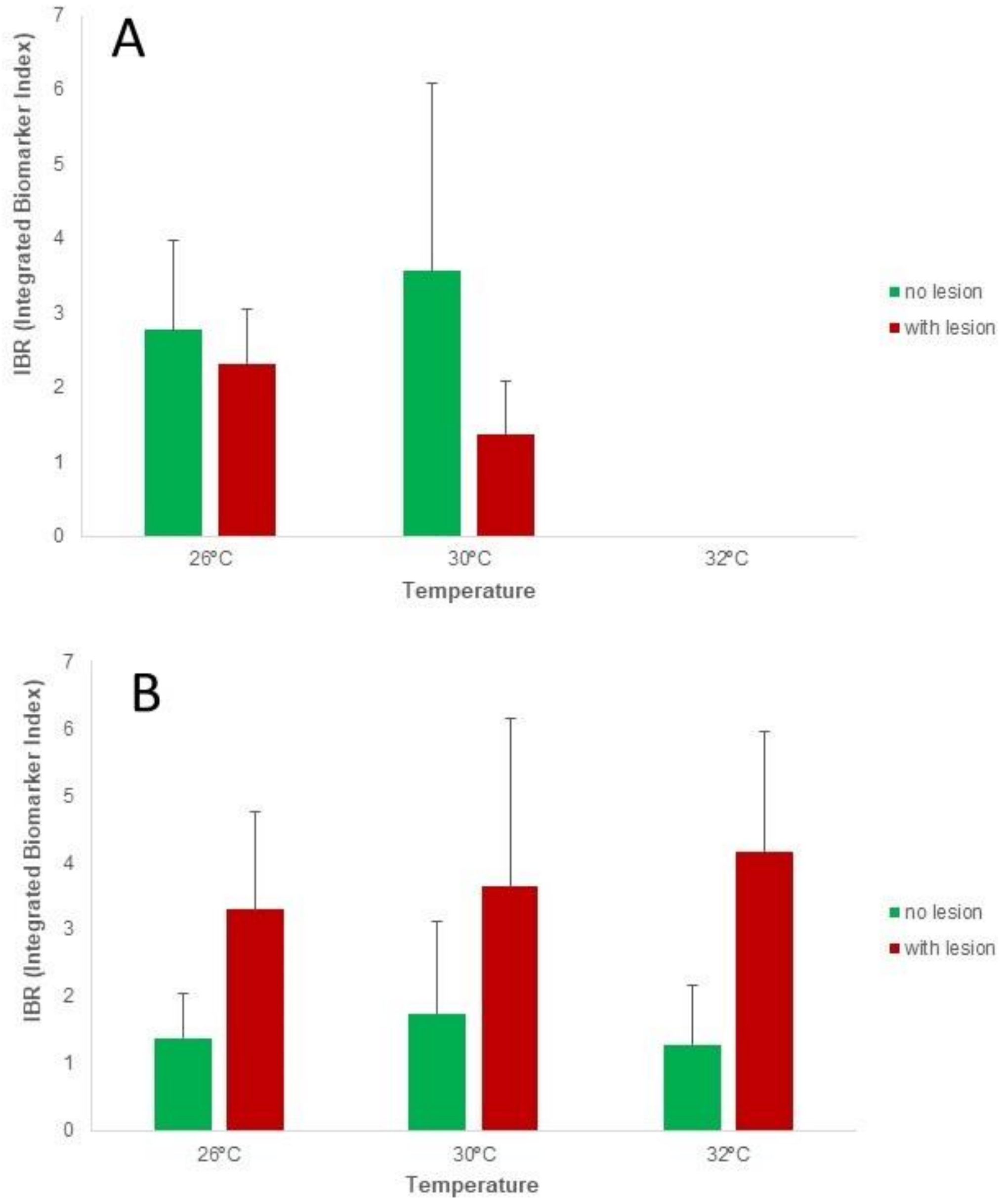

Fig. SM12 IBR values (mean+sd) for A) Thermosensitive species (which died at 32 ºC, *A. tenuis*, *E. lamellosa*, *M. capricornis* BM and GM) and B) Thermotolerant species (which survived at 32 ºC, *G. fasciculares*, *P contigua* and *T. reniformis*). Note: the absence of values at 32 ºC means that the group of species died at that temperature.
